# Age- and sex-dependent heterogeneity during LPS-induced murine acute lung injury

**DOI:** 10.1186/s12979-026-00592-6

**Published:** 2026-08-26

**Authors:** Chantal Crispens, Annett Wilken-Schmitz, Valentin Dern, Abd Rahman Rahimi, Enrico Madau, Nadine Chiara Frigger, Josefine Jakob, Björn Häupl, Thomas Oellerich, Andreas Weigert, Steven R. Talbot, Kai Zacharowski, Ulrike Heinicke, Andreas von Knethen

**Affiliations:** 1 Department of Anesthesiology, Intensive Care Medicine and Pain Therapy, Goethe University Frankfurt, University Hospital Frankfurt, Theodor-Stern-Kai 7, Frankfurt Am Main, 60590 Germany; 2 Frankfurt Cancer Institute, Theodor-Stern-Kai 7, Frankfurt, 60590 Germany; 3 Department of Hematology and Oncology, Goethe University Frankfurt, University Hospital, Theodor-Stern-Kai 7, Frankfurt, 60590 Germany; 4 German Cancer Consortium (DKTK), a Partnership Between DKFZ and UCT Frankfurt-Marburg, Frankfurt Am Main, Germany; 5 Goethe University Frankfurt, Faculty of Medicine, Institute of Biochemistry I, Frankfurt, 60590 Germany; 6 Department for Immunity of Inflammation, Mannheim Institute for Innate Immunoscience (MI3), Medical Faculty Mannheim, Heidelberg University, Mannheim, Germany; 7 Hannover Medical School, Institute for Laboratory Animal Science, Carl-Neuberg-Straße 1, Hannover, 30625 Germany; 8 Institute of Medical Statistics, RWTH Aachen University Hospital, Aachen, Germany

**Keywords:** ALI, ARDS, MICU, Inflammation, Ageing, Sex- and age-specific differences, Mouse model, Immune response

## Abstract

Acute respiratory distress syndrome (ARDS) remains difficult to treat due to its heterogeneous etiology. In this study, a mouse model of intratracheal lipopolysaccharide (LPS) instillation was used to investigate age- and sex-dependent immune responses. After six hours of LPS exposure and one hour of mechanical ventilation, immune cell subtypes as well as mRNA and protein expression were analyzed. Aged mice exhibited a basal pro-inflammatory immune signature already under sham conditions, whereas young mice displayed a more balanced response with parallel pro- and anti-inflammatory mechanisms. Notably, aged female LPS treated mice exhibited an exclusively pro-inflammatory response. UMAP analysis revealed distinct clustering according to age, sex, and treatment, while aged male mice formed isolated clusters. These findings highlight the importance of considering age- and sex-specific therapeutic strategies for ARDS treatment.

**Graphical Abstract:**

**Figure:**
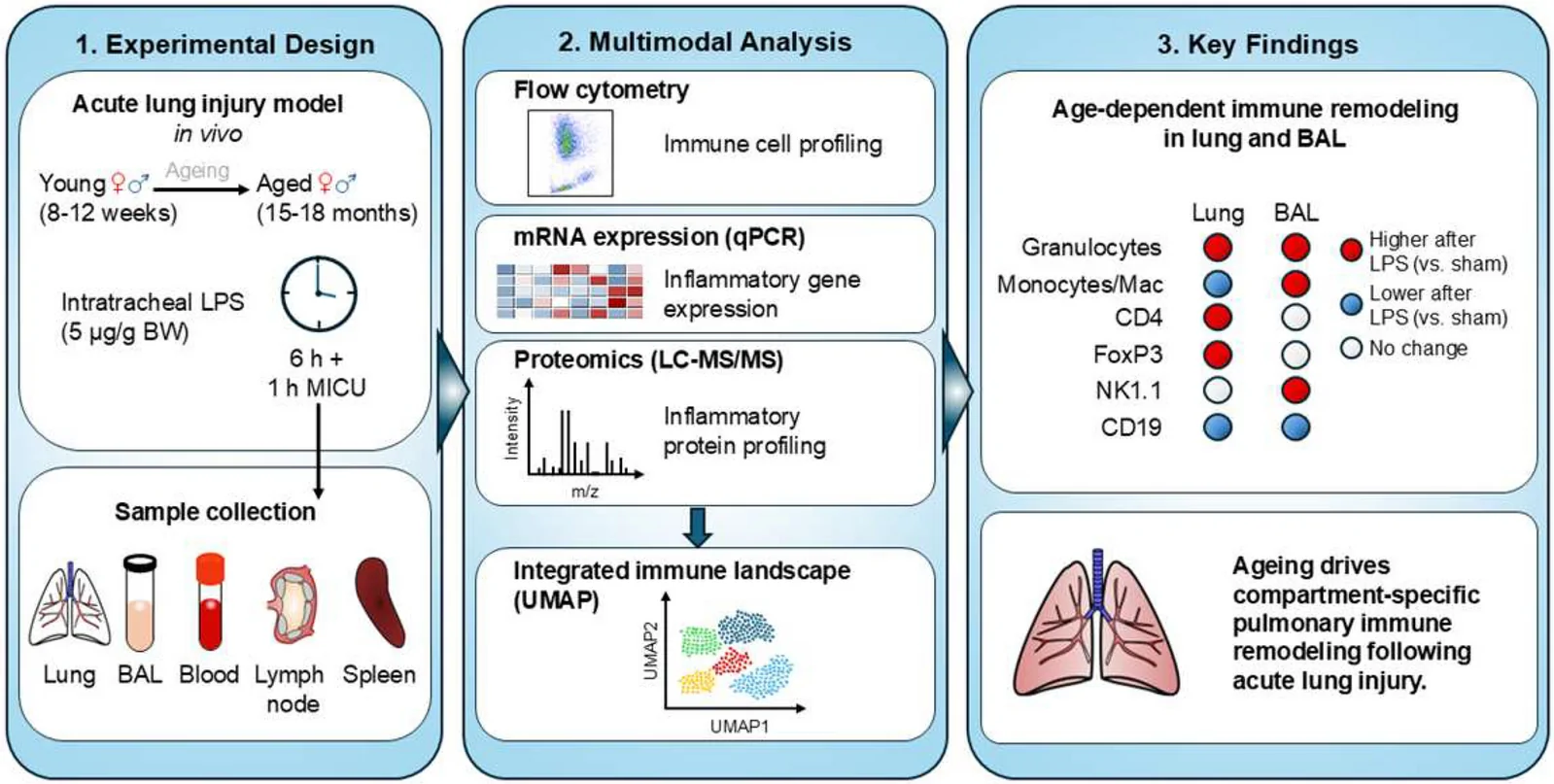

**Supplementary Information:**

The online version contains supplementary material available at https://doi.org/10.1186/s12979-026-00592-6.

## Introduction

Patients with acute respiratory distress syndrome (ARDS) develop acute hypoxemic respiratory failure characterized by non-cardiogenic pulmonary edema, impaired gas exchange, and the need for mechanical ventilation [1]. Since its initial description in 1967 [2], the clinical definition of ARDS has evolved through the Berlin definition [3] and the subsequent Kigali modification [4], enabling classification into mild, moderate, and severe disease according to the degree of hypoxemia (PaO₂/FiO₂ 201–300, 101–200, and ≤ 100 mmHg, respectively). Despite substantial advances in supportive intensive care and renewed interest following the COVID-19 pandemic [5, 6], treatment remains largely supportive, as no causal therapy is currently available for this biologically heterogeneous syndrome [7]. Consequently, ARDS continues to be associated with mortality rates approaching 40%, representing a major unmet clinical need [8].

One promising strategy to improve clinical management is the identification of biologically and clinically meaningful patient subtypes and endotypes. These may respond differently to therapeutic interventions. Such stratification forms the basis of precision medicine and is increasingly recognized as an important prerequisite for individualized treatment approaches in ARDS [9–11]. Ageing is one of the strongest determinants of disease susceptibility and outcome, with elderly patients exhibiting substantially higher mortality than younger individuals [12]. Age-associated remodeling of the epigenetic landscape contributes to immune senescence and profoundly alters both innate and adaptive immune responses [13–16]. These changes are accompanied by a shift from lymphoid toward myeloid hematopoiesis, resulting in chronic low-grade inflammation, commonly referred to as inflammageing [17–21].

Besides ageing, biological sex represents another major determinant of immune function. Sex hormones, including estrogens, androgens, and progesterone, regulate immune-cell differentiation, activation, cytokine production, and inflammatory signaling [22–25]. Consequently, age and sex should not be considered independently but rather as interacting biological variables that together shape immune responses [13, 22, 26–28]. Although increasing evidence supports age- and sex-dependent differences in immunity, the mechanisms by which these factors influence pulmonary inflammation and its resolution during acute lung injury remain incompletely understood.

To address this question, we investigated age- and sex-dependent pulmonary immune responses during experimental acute lung injury. In addition to classical pro-inflammatory cytokines, we analyzed regulatory mediators involved in inflammatory resolution, macrophage polarization, oxidative stress, extracellular-matrix remodeling, and vascular integrity, thereby providing a comprehensive characterization of pulmonary inflammatory regulation [29–39]. For this purpose, we employed an established murine model of lipopolysaccharide (LPS)-induced acute lung injury using intratracheal LPS administration. This model enabled us to investigate age- and sex-dependent differences in local and systemic immune responses under standardized experimental conditions. Although biological differences associated with ageing and sex have long been recognized, their integration into precision-medicine concepts for ARDS remains incomplete [13, 26, 28, 40–44]. Using young (8–12 weeks) and aged (15–18 months) female and male mice, we characterized immune-cell populations by multicolor flow cytometry together with pulmonary mRNA and protein expression profiles. To integrate these multidimensional datasets, we performed unsupervised dimensionality reduction using Uniform Manifold Approximation and Projection (UMAP), enabling the identification of age- and sex-dependent immune signatures associated with experimental LPS-induced lung injury. These integrated analyses provide a framework for understanding biological diversity in experimental ARDS and may contribute to the development of future precision-medicine strategies.

## Results

### Integrated flow cytometric analysis identifies compartment-, age-, and sex-dependent immune signatures following LPS challenge

**Fig. 1:**
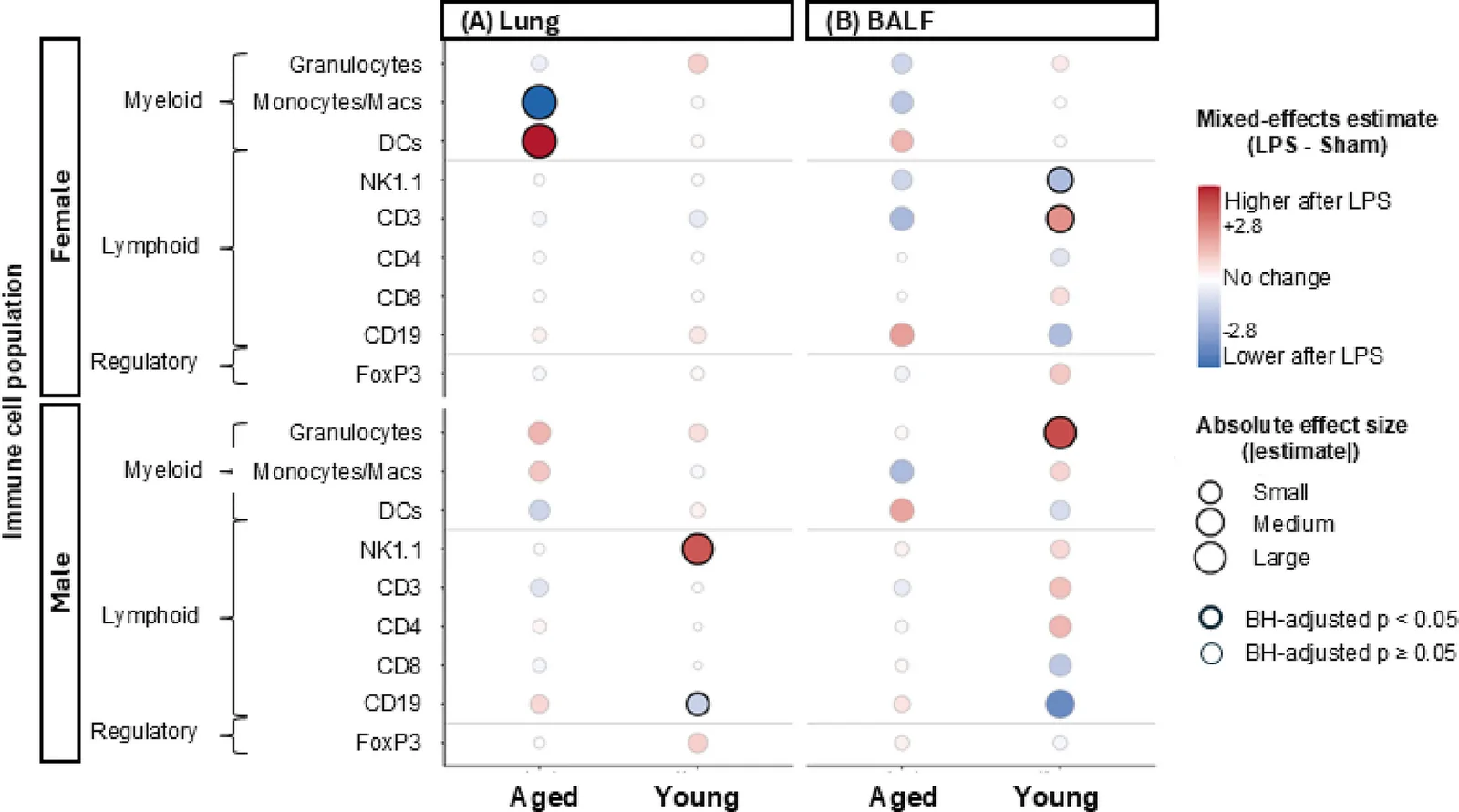
Integrated flow cytometric analysis identifies age- and sex-dependent immune signatures in the lung and bronchoalveolar lavage fluid following LPS challenge. Flow-cytometric immune-cell frequencies were analyzed using linear mixed-effects models including age, sex, treatment (LPS vs. sham), and tissue compartment as fixed effects and mouse as a random intercept. Estimated treatment effects (LPS − sham) are displayed as bubble heatmaps for (A) lung and (B) bronchoalveolar lavage fluid (BALF). Rows represent the analyzed immune-cell populations grouped into myeloid (granulocytes, monocytes/macrophages, dendritic cells), lymphoid (NK1.1⁺, CD3⁺, CD4⁺, CD8⁺ and CD19⁺ cells), and regulatory (FoxP3⁺ Tregs) compartments. Columns represent aged and young animals separated by sex. Bubble color indicates the estimated direction and magnitude of the LPS-induced response relative to the corresponding sham controls (red = higher after LPS; blue = lower after LPS). Bubble size reflects the absolute effect size (|estimate|). Bubbles outlined in black indicate statistically significant treatment effects after Benjamini–Hochberg correction (BH-adjusted *p* < 0.05), whereas circles without an outline indicate non-significant differences. Immune-cell frequencies were determined by multicolor flow cytometry using the gating strategy shown in Supplementary Fig. S2. Frequencies were calculated relative to the respective parent population as described in the Methods. The corresponding bar graphs displaying the underlying immune-cell frequencies for all analyzed tissues are provided in Supplementary Figs. S3–S8

To comprehensively characterize the immune response to intratracheal LPS administration, flow-cytometric data were analyzed using linear mixed-effects models including age, sex, treatment, and tissue compartment as fixed effects and mouse as a random intercept. Estimated treatment effects are summarized as bubble heatmaps, in which color indicates the direction of the LPS-induced response, bubble size reflects the magnitude of the estimated effect, and black outlines denote statistically significant differences after Benjamini–Hochberg correction (Fig. 1A, B). The underlying flow-cytometric frequencies for all immune-cell populations are shown in Supplementary Figs. S3–S8.

In the lung, myeloid immune-cell populations displayed pronounced age- and sex-dependent response patterns (Fig. 1A). Young female mice exhibited the strongest positive granulocyte response following LPS treatment, whereas aged females showed comparatively attenuated granulocyte responses. In contrast, dendritic cells displayed the most prominent positive response in aged females, while monocyte/macrophage frequencies were predominantly reduced. Within the lymphoid compartment, treatment-associated alterations were generally less pronounced, although NK1.1⁺ cells showed a marked response in young males. FoxP3⁺ regulatory T cells exhibited only moderate changes across experimental groups.

The immune signature observed in BALF differed substantially from that detected in lung tissue (Fig. 1B). Granulocyte responses were most pronounced in young males, whereas macrophage frequencies generally decreased following LPS treatment. Dendritic cells again showed preferential responses in aged animals, while lymphoid populations demonstrated distinct but overall less extensive treatment-associated alterations. These findings demonstrate that pulmonary immune responses differ considerably between lung tissue and the alveolar compartment.

Collectively, the mixed-effects analysis demonstrated distinct compartment-, age-, and sex-dependent immune signatures in response to intratracheal LPS challenge.

### Systemic immune signatures differ from pulmonary immune responses

To determine whether pulmonary immune alterations were accompanied by systemic immune responses, immune-cell populations were additionally analyzed in peripheral blood, mediastinal lymph nodes, and spleen using the same mixed-effects modelling approach (Supplementary Fig. S9). The corresponding flow-cytometric frequencies underlying these analyses are provided in Supplementary Figs. S10–S17.

Compared with the pulmonary compartment, systemic immune responses exhibited distinct compartment-specific patterns. In blood, treatment-associated changes were most evident within myeloid immune-cell populations and differed predominantly between female and male mice. In particular, granulocyte, monocyte/macrophage, and dendritic-cell responses demonstrated pronounced sex-dependent alterations, whereas lymphoid populations and FoxP3⁺ regulatory T cells showed comparatively modest changes.

The mediastinal lymph nodes displayed a distinct regional immune signature characterized by pronounced alterations in dendritic cells together with more moderate changes in granulocyte and monocyte/macrophage populations. Lymphoid immune-cell populations and FoxP3⁺ regulatory T cells likewise demonstrated selective age- and sex-dependent treatment responses.

In the spleen, LPS-induced alterations were less widespread and were predominantly confined to myeloid immune-cell populations. In contrast, lymphoid populations and FoxP3⁺ regulatory T cells remained comparatively stable across experimental groups.

Collectively, these findings demonstrate that intratracheal LPS administration induces compartment-specific immune signatures extending beyond the lung. However, the magnitude and direction of these responses differ substantially between pulmonary and systemic immune compartments, emphasizing the complex influence of age and biological sex on the organization of the inflammatory response.

### mRNA expression of immune-regulatory and oxidative stress-related genes of LPS-stimulated lung tissue

**Fig. 2:**
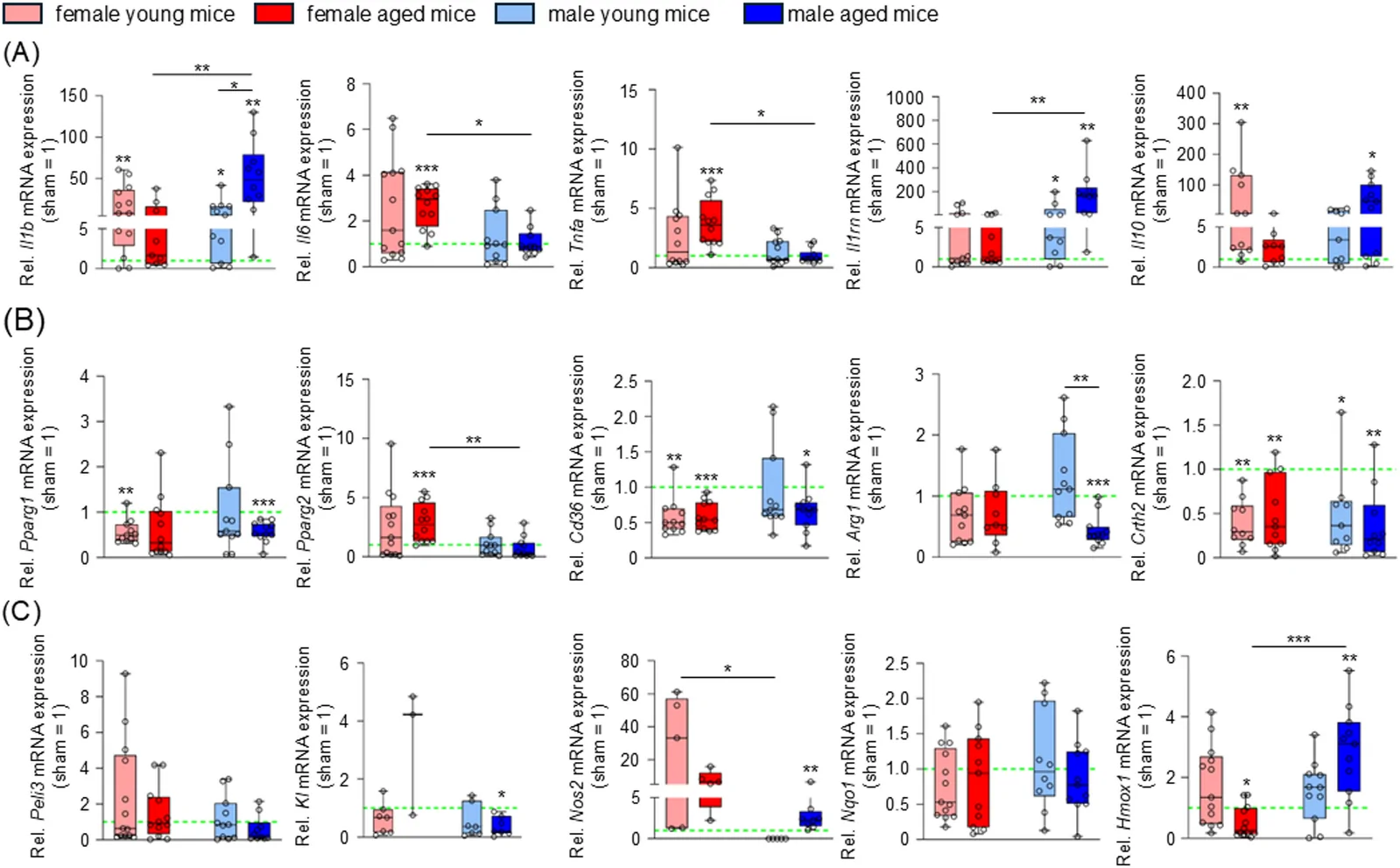
mRNA expression profiles of young and aged female as well as male lung tissue. 6 h after LPS stimulation + 1 h MICU, normalized to the respective sham controls (= 1). The horizontal green dotted line represents the corresponding sham groups. **A** Relative mRNA expression of the depicted pro-inflammatory mediators *Il1b* (left panel), *Il6* (second panel), and *Tnfa* (hird panel), as well as the anti-inflammatory factors *Il1 receptor antagonist* (*Il1rn*, fourth panel) and *Il10* (right panel). **B** Relative mRNA expression of factors established in contributing to macrophage polarization from M1 to M2 and vice versa. I.e., relative mRNA expression of *Pparg1* (left panel) and 2 (second panel), *Cd36* (third panel), *Arg1* (fourth panel), and *Crth2* (right panel). **C** Relative mRNA expression of proteins that are involved in immune signalling as well as regulated upon oxidative stress. Thus, we examined mRNA expression of *Peli3* (first panel), *Kl* (second panel) and *Nos2* (third panel) as a further pro-inflammatory marker, as well as the redox-regulated genes *Nqo1* (fourth panel) and *Hmox1* (right panel). Data are shown as whisker box plots providing medians, IQR, Q1, Q3, minimum, and maximum data values. Statistical differences were assessed via the Kruskal–Wallis test for non-normally distributed data and the ordinary one-way ANOVA when normally distributed. Normalized samples were calculated as a fold of sham using the Wilcoxon one-sample signed rank test. *p < 0.05, **p < 0.01, ***p < 0.001. Light red, young female; blazing red, aged female; light blue, young male; royal blue, aged male mice; dotted green line (= sham) was set as 1. LPS, lipopolysaccharide; MICU, mouse intensive care unit

To determine whether the compartment-specific immune signatures identified by flow cytometry were accompanied by molecular alterations within the lung, we next analyzed pulmonary mRNA expression of inflammatory, regulatory, and oxidative stress-associated genes (Fig. 2A-C). We first analyzed classical pro- vs. anti-inflammatory cytokines. LPS significantly induced *Il1b* mRNA expression in all groups except aged female mice. While Il1b responses were comparable between young females, aged females, and young males, aged male mice exhibited significantly higher *Il1b* expression compared with both aged females and young males, indicating an exaggerated pro-inflammatory response in this group **(**Fig. 2A**)**. In contrast, *Il6* and *Tnfa* expression displayed a striking sex- and age-specific pattern. Both cytokines were robustly induced exclusively in aged female mice, whereas no significant induction was detected in young females or in male mice of either age. Accordingly, *Il6* and *Tnfa* mRNA levels were significantly higher in aged females compared with aged males following LPS challenge, highlighting a female-specific inflammatory transcriptional program in aged lungs (Fig. 2A). We next assessed anti-inflammatory mediators. Expression of the Il1 receptor antagonist (*Il1rn*) was selectively induced in male mice, whereas female mice failed to mount a comparable response. *Il1rn* expression was highest in aged male mice, exceeding levels observed in aged females. *Il10* mRNA expression showed only modest regulation; however, young female and aged male mice displayed a mild but significant induction compared with their respective sham controls (Fig. 2A).

To further explore macrophage polarization states, we determined the expression of the nuclear receptor *Pparg* [31] and its isoforms. Interestingly, *Pparg1* and *2* exhibited opposing regulation. *Pparg1* expression was significantly reduced in young female and aged male mice, while remaining unchanged in aged females and young males. In contrast, Pparg2 was selectively upregulated in aged female mice, resulting in significantly higher expression compared with aged males (Fig. 2B). Consistent with reduced *Pparg1* activity, expression of the *Pparg* target gene *Cd36* was downregulated in young and aged females as well as in aged males, whereas young male mice were unaffected. Similarly, *Arg1* expression was selectively suppressed in aged male animals and showed a non-significant downward trend in female mice in response to LPS compared to sham mice (Fig. 2B). This downregulation of *Arg1* in aged male mice was also evident compared with young males, pointing to an age-dependent control in male animals (Fig. 2B).

Finally, we examined genes involved in immune signaling and oxidative stress responses (Fig. 2B/C). T_h_2-associated marker *Crth2* was uniformly downregulated in all LPS-treated groups, independent of sex or age (Fig. 2B). Expression of *Peli3*, a negative regulator of TLR4 signaling, remained unchanged across all experimental groups. In contrast, the longevity-associated gene *Klotho* (*Kl*) [45] was selectively downregulated in aged male LPS-treated mice (Fig. 2C). Analysis of inducible nitric oxide synthase (*Nos2*) revealed a pronounced induction in aged male mice, whereas female mice showed a moderate, non-significant increase. Notably, *Nos2* expression was higher in young females compared with young males, indicating sex-dependent regulation of nitric oxide signaling (Fig. 2C). Assessment of antioxidant defense pathways revealed no significant regulation of the *Nrf2* target gene *Nqo1* (Fig. 2C). However, expression of *Hmox1* displayed marked age- and sex-dependent regulation. Aged female mice showed a significant reduction of *Hmox1* expression following LPS challenge, whereas aged male mice exhibited a robust induction. *Hmox1* levels in aged males were significantly higher than in aged females, while remaining unchanged in young mice of either sex (Fig. 2C).

Conclusively, these data pointed to a balanced mRNA expression of pro- vs. anti-inflammatory mediators in young LPS-treated mice, favoring an adequate immune response, which was different in aged animals. Thus, aged females were characterized by a dominant expression of pro-inflammatory genes without a compensatory expression of anti-inflammatory genes, whereas in aged males, there was robust expression of pro-inflammatory markers as well as high expression of anti-inflammatory proteins, which was putatively associated with a cytokine storm and an inappropriate immune reaction.

### Age- and sex-dependent remodeling of the lung proteome following LPS challenge

**Fig. 3:**
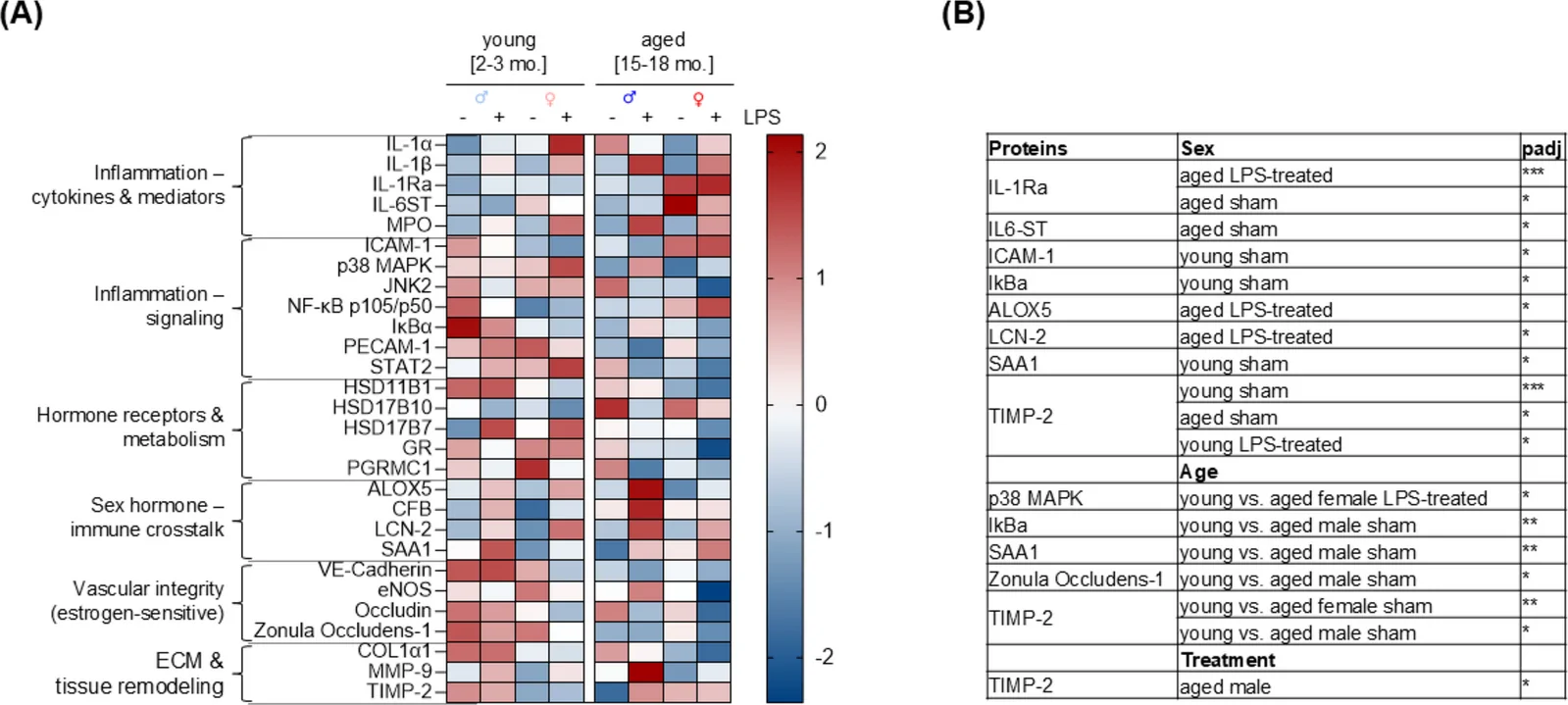
Sex- and age-specific regulation of hormone–immune crosstalk proteins following LPS-induced acute lung injury. **A** Heatmap showing z-score–normalized log₂ fold changes (LPS vs. Sham) for 28 hormone–immune crosstalk proteins across four sex- and age-defined groups at 6 h + 1 h MICU post-LPS challenge. Proteins are grouped into six functional categories reflecting roles in inflammatory signalling, hormone receptor pathways, and vascular integrity. Red indicates upregulation; blue indicates downregulation. ♂ male young; ♂, male aged; ♀, female young; ♀, female aged; mo., months. **B** Proteins derived from (**A**) showing significant changes for sex, age, and treatment. Adjusted p-values are shown. *padj < 0.05, **p < 0.01, ***p < 0.001. LPS, lipopolysaccharide; MICU, mouse intensive care unit

The pronounced age- and sex-specific differences observed at the cellular and transcriptional levels indicate that the pulmonary immune response to LPS is governed by complex regulatory mechanisms that extend gene expression alone. To capture these regulatory layers and account for post-transcriptional and post-translational control, we next performed an unbiased proteomic analysis of lung tissue using ultrasensitive LC–MS/MS. Considering the potential influence of sex- and age-associated factors, including hormonal regulation on immune signaling, we aimed to define age and sex-dependent proteomic signatures following LPS challenge. This approach enabled the identification of molecular pathways that may mechanistically link the observed differences in immune cell composition and transcriptional responses across experimental groups. We screened the total proteome, consisting of 4634 proteins, for proteins established in hormone-inflammation crosstalk. In the IL-1 system protein expression of IL-1α and IL-1β showed a sex-specific increase in LPS-treated young and aged female mice (Fig. 3A), which was compensated by expression of its antagonist IL-1Ra, which however was already upregulated in aged female sham animals and stayed low in aged male sham animals (Fig. 3B). Its LPS-dependent substantial downregulation in aged males compared to aged females further corroborated a sex-specific regulation of expression (Fig. 3B). Protein expression of IL6ST was also found to be sex-specific with low expression in male mice and an enhanced expression in female mice, leading to a substantial enhanced expression in aged female sham mice compared to their male counterparts (Fig. 3B). Myeloperoxidase (MPO) expression was sex-independently upregulated in LPS-treated mice of either age (Fig. 3A). Among proteins established in inflammatory signaling we identified ICAM-1 and IκBα to be expressed in young male sham mice compared to their female counterparts (Fig. 3B). Additionally, expression of IκBα was significantly reduced in aged male sham mice (Fig. 3B). The expression of NF-κB p105/p50 was also high in young male animals and low in their female equivalents (Fig. 3A). In aged animals, this picture was different, showing NF-κB p105/p50 upregulation in females and a decreased expression in males. Moreover, there was an age-dependent regulation of p38MAPK, showing a substantial reduction in aged female LPS-treated mice compared to their young female counterparts (Fig. 3B), and furthermore of JNK2, PECAM-1 and STAT2, which all were expressed in young animals of either sex (Fig. 3A).

Focusing on enzymes involved in the synthesis of steroid hormones (HDS11B1, HSD17B7, −10) and receptors mediating their signaling (GR, PGRMC1), we found an LPS-dependent induction of HSD17B7 in young mice of either sex, whereas HSD11B1 was mainly expressed in male animals of both ages and HSD17B10 was predominantly expressed in lungs of aged sham-mice (Fig. 3A). Further age-dependent control was observed in GR protein expression, mostly detected in young animals and in PGRMC1 primarily found in young sham mice (Fig. 3A).

ALOX5, belonging to proteins established in sex hormone immune crosstalk, was induced by LPS in young male and female mice as well as aged male animals (Fig. 3A), but in contrast significantly downregulated in aged females (Fig. 3B). The complement factor B (CFB) was expressed in aged animals, however showing the highest expression in aged male LPS-treated mice (Fig. 3A). LCN-2 was induced by LPS in all animals (Fig. 3A), conversely showing a significant higher expression in aged male LPS-treated mice compared to their female counterparts (Fig. 3B). The acute phase protein SAA1 was induced by LPS. Interestingly, there was a difference in its expression pattern in sham animals, showing a higher expression of SAA1 in young males than young females, suggesting a sex- and age-dependent regulation, which is further corroborated in aged male sham mice, with a significantly decreased expression compared to their young male counterparts (Fig. 3B).

To determine the vascular integrity, we analyzed the expression of proteins such as VE-cadherin, eNOS, occludin and Zonula Occludens 1 (ZO-1). All of them were expressed in young animals, indicating an age-dependent regulation (Fig. 3A), which was further shown by a significantly lower ZO-1 expression in aged male sham mice compared to their young counterparts (Fig. 3B).

Finally, centering around factors important for the synthesis of extracellular matrix and tissue remodeling, we determined the expression of collagen COL1a1, the matrix metalloproteinase MMP-9, and its inhibitor TIMP-2. In male mice, COL1a1 was expressed independently from LPS-treatment. MMP-9 was induced by LPS in all animals. Its inhibitor TIMP-2, showed a tight regulation, involving sex, age, and treatment. Significantly higher TIMP-2 expression in young male sham mice compared to the female counterparts revealed a sex-specific regulation of expression (Fig. 3B). Interestingly, this pattern of expression was inverted in aged animals, outlined by high expression in female and low expression in male sham animals. Moreover, we also observed an age-related expression of TIMP-2 with low expression in young and high expression in aged sham animals (Fig. 3B). Lastly, TIMP-2 expression was induced by LPS in aged male mice (Fig. 3B).

Proteins being active in inflammation, linking hormones to metabolism and immune responses, guaranteeing vascular integrity as well as tissue remodeling, were differentially regulated depending on sex, age, and treatment (Fig. 3A). Despite these obvious variations, not all analyzed proteins were significantly regulated (Fig. 3B, Tables S9 to S11). Conclusively, these data support the notion that young mice were characterized by a balanced and effective immune response in the lung, which is different in aged animals, where we found an inflammatory focus already in sham females.

### Combination of analyzed immune cell subpopulations, proteins, and genes

**Fig. 4:**
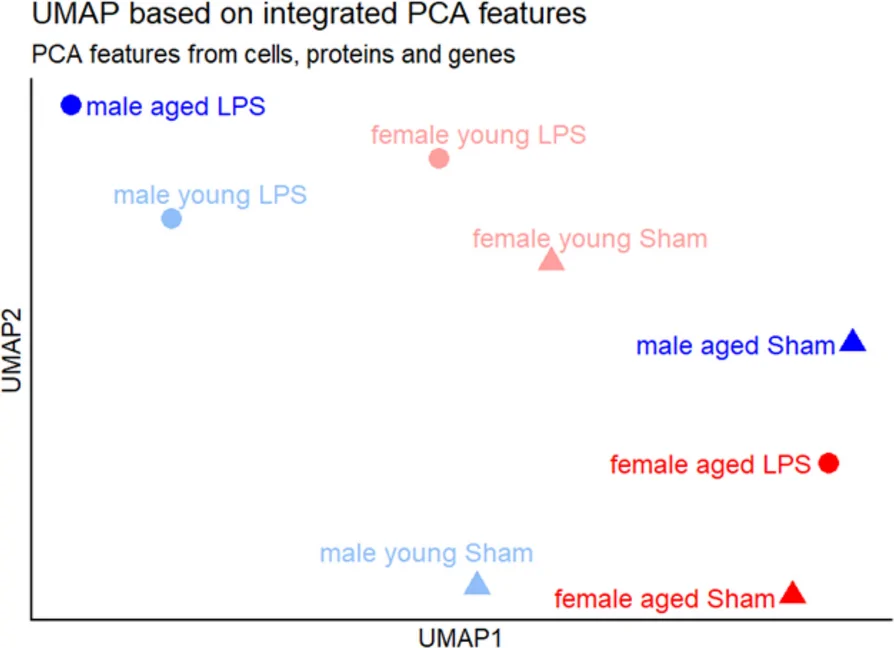
Integrated multidimensional analysis identifies age- and sex-dependent pulmonary immune signatures. UMAP showing a combination of z-score-normalized data for pulmonary immune cell subtypes, mRNA expression of immune response relevant genes, and proteins of young (8–12 weeks) and aged (15–18 months) female and male animals (LPS vs. Sham). UMAP showed sex and age-specific differences, but a relative clustering according to sham and LPS treatment, apart from aged male mice. These also differed treatment dependently. Light red triangle, female young sham mice; light red filled circle, female young LPS animals; blazing red triangle, female aged sham mice; blazing red filled circles, female aged LPS animals; light blue triangle, young male sham mice, light blue filled circle, young male LPS animals; royal blue triangle, male aged sham mice; royal blue filled circle, male aged LPS animals. LPS, lipopolysaccharide; UMAP, uniform manifold approximation and projection for dimension reduction

To link our data about differences in immune cell subpopulations in the lung, including myeloid, lymphoid, and T_reg_ panels of female, male, young, and aged animals (Fig. 1A-B) with our lung mRNA (Fig. 2A-C), and lung protein data (Fig. 3A), we performed principal component analyses [46] (PCA, Figs. S17A-C) of calculated z-scores (Tables S11 to S12), finally combined to an uniform manifold approximation and projection for dimension reduction (UMAP) picture (Fig. 4) [47].

PCAs of the lung immune cell subpopulations, which are based on flow cytometric results (Fig. S17A), lung mRNA expression determined by qPCR (Fig. S17B), and lung protein expression analyzed by MS (Fig. S17C), considered separately, already revealed differences related to sex and age. Integrating these into a UMAP view (Fig. 4), revealed no general effect of age on the response to LPS, since only male, but not female young and aged mice clustered further apart upon LPS compared treatment when compared with their sham counterparts (Fig. 4). Focusing on sex-dependent divergences, the response to LPS was much more pronounced in young and aged male sham groups compared to female mice, with young and aged male mice following LPS treatment clustering closely together (Fig. 4). Interestingly, the sham groups of young male and aged female, as well as young female and aged male mice were clustering closely together (Fig. 4).

## Discussion

Acute respiratory distress syndrome (ARDS) remains one of the leading causes of mortality in critically ill patients despite considerable advances in supportive intensive care medicine. One major reason for the limited success of targeted therapies is the pronounced biological heterogeneity of ARDS. Patients fulfilling identical clinical diagnostic criteria exhibit substantial differences in inflammatory activation, immune-cell composition, and molecular signaling pathways, suggesting that ARDS comprises multiple biological endotypes rather than a single disease entity. Consequently, recent research has shifted towards precision medicine approaches that aim to integrate cellular and molecular biomarkers into patient stratification to enable individualized therapeutic interventions [1, 7–11, 48–51].

Among the biological factors contributing to this heterogeneity, ageing represents one of the strongest determinants of ARDS susceptibility and outcome. Rather than simply impairing immune function, biological ageing induces profound remodeling of both innate and adaptive immunity through alterations in hematopoietic stem-cell biology, myeloid differentiation, inflammatory signaling, tissue repair, and immune regulation. These processes collectively contribute to the chronic low-grade inflammatory state termed *inflammageing*, which substantially modifies host responses to acute inflammatory injury [14–21, 29, 30, 52, 53]. Biological sex represents an additional major determinant of immune regulation. Sex hormones, sex chromosome complement, and epigenetic mechanisms influence leukocyte differentiation, cytokine production, adaptive immune responses, and susceptibility to inflammatory diseases. Consequently, age and sex should not be considered independent variables but interacting biological modifiers of pulmonary inflammation [13, 22–28, 35–44].

Using a previously established mouse intensive care unit (MICU) model of intratracheal LPS-induced acute lung injury [54, 55], we combined multicolor flow cytometry, pulmonary gene-expression analysis, unbiased LC–MS/MS-based proteomics, and multidimensional integration by UMAP to comprehensively characterize age- and sex-dependent inflammatory responses. This integrated multi-layered approach demonstrated that biological ageing does not induce a single inflammatory phenotype but instead reorganizes pulmonary immune responses at the cellular, transcriptional, and proteomic levels. Moreover, these alterations differed substantially between pulmonary and systemic compartments, further emphasizing that inflammatory responses during acute lung injury are highly context dependent. Together, these findings provide experimental support for current concepts proposing biologically distinct ARDS endotypes and highlight the importance of integrating multiple biological data layers when investigating inflammatory heterogeneity [9–11, 48–51, 56–60].

A particular strength of the present study is the comprehensive evaluation of immune-cell composition across lung tissue, bronchoalveolar lavage fluid (BALF), blood, mediastinal lymph nodes, and spleen using linear mixed-effects modelling. While previous experimental studies have frequently analyzed individual tissues or isolated immune-cell populations, our approach simultaneously accounted for age, sex, treatment, and anatomical compartment while correcting for inter-individual biological variability. This strategy revealed pronounced compartment-specific immune responses following intratracheal LPS administration and demonstrated that pulmonary inflammation cannot be adequately described by analyses confined to a single anatomical site. Instead, inflammatory responses represent coordinated multicompartment immune processes involving distinct local and systemic immune adaptations [56, 61–63].

Within the pulmonary compartment, biological ageing altered the overall organization of inflammatory responses rather than uniformly enhancing or suppressing individual immune-cell populations. Although granulocytes, monocyte/macrophage populations, dendritic cells, lymphocytes, and regulatory T cells all responded to LPS challenge, their relative responses differed according to age, sex, and tissue compartment. Particularly notable was the divergent regulation of myeloid populations in aged animals, suggesting that ageing primarily reshapes immune-cell organization instead of inducing generalized immune dysfunction. This interpretation agrees with recent concepts describing inflammageing as a dynamic and context-dependent remodeling of immune function rather than a simple deterioration of host defense [16–21, 29, 52, 53].

Importantly, alterations in immune-cell frequencies should not automatically be interpreted as evidence of impaired immune competence. Ageing affects multiple biological processes that collectively determine immune-cell composition within inflamed tissues, including hematopoietic output, chemokine responsiveness, endothelial transmigration, tissue retention, apoptosis, and inflammatory resolution. Consequently, differences in leukocyte frequencies may reflect altered migration kinetics or compartmental redistribution rather than quantitative immune deficiency. The marked differences observed between lung tissue and BALF despite identical inflammatory stimulation strongly support this concept and underline the importance of compartment-specific immune analyses when studying pulmonary inflammation [16–21, 61, 62].

Neutrophils are indispensable mediators of early pulmonary host defense but simultaneously represent major contributors to tissue injury through the release of reactive oxygen species, proteases, inflammatory cytokines, and neutrophil extracellular traps. Oxidative stress is increasingly recognized as a central mechanism linking excessive innate immune activation with epithelial and endothelial injury during ARDS [33, 34]. Although functional neutrophil responses were not directly investigated in the present study, the observed age-dependent granulocyte response patterns are compatible with the concept that ageing primarily modifies neutrophil function and inflammatory regulation rather than merely increasing leukocyte recruitment. Future studies should therefore combine quantitative immune-cell profiling with functional analyses of neutrophil activation and resolution pathways.

Finally, our findings demonstrate that biological sex substantially modifies age-associated inflammatory responses. Rather than acting as an isolated biological variable, sex interacted with ageing to shape pulmonary immune organization across multiple immune-cell populations. These observations are consistent with growing evidence demonstrating profound sex-dependent differences in innate immunity, adaptive immune responses, inflammatory diseases, and clinical outcomes in critically ill patients [13, 22–28, 35–38, 41–43]. Incorporating both age and sex into experimental and translational studies will therefore be essential for improving biological interpretation and facilitating the development of personalized therapeutic approaches for ARDS.

The transcriptional analyses further demonstrate that pulmonary inflammation during acute lung injury is not solely determined by changes in immune-cell composition but is accompanied by profound alterations in inflammatory gene programs. Rather than identifying isolated changes in individual cytokines, our data indicate coordinated remodeling of pro-inflammatory, regulatory, and tissue-protective pathways that differs according to age and biological sex. This observation is consistent with current concepts proposing that inflammatory responses are orchestrated through dynamic transcriptional networks rather than individual signaling molecules [9, 11, 29, 30].

The altered expression of *Il1b*, *Il6*, and *Tnfa* observed in the present study highlights the central role of innate inflammatory signaling during experimental acute lung injury. IL-1 and IL-6 are well-established mediators of pulmonary inflammation that amplify leukocyte recruitment, endothelial activation, and cytokine production, whereas TNF-α initiates multiple downstream inflammatory cascades. However, increasing evidence indicates that ageing does not simply enhance cytokine production but instead modifies the magnitude, timing, and coordination of inflammatory responses. Consequently, differences in cytokine expression between young and aged animals are likely to reflect age-dependent immune remodeling rather than exaggerated inflammation alone [29, 30, 52, 64].

Importantly, pulmonary inflammation is simultaneously regulated by endogenous anti-inflammatory mechanisms. In this context, Il-1Ra and IL-10 constitute essential components of inflammatory resolution by limiting excessive cytokine signaling and protecting surrounding tissue from collateral damage. Rather than representing simple anti-inflammatory markers, both mediators contribute to the fine regulation of immune homeostasis. The coordinated evaluation of pro- and anti-inflammatory genes in the present study therefore provides a more comprehensive assessment of pulmonary immune regulation than analysis of individual cytokines alone and illustrates that inflammatory activity should be interpreted as the balance between activating and regulatory pathways rather than the expression of single mediators [30, 65, 66].

Beyond classical cytokines, our data further indicate age-dependent alterations in transcriptional programs associated with macrophage differentiation and immunometabolic regulation. PPARγ represents a key transcriptional regulator of alternative macrophage activation and contributes to inflammatory resolution by promoting lipid metabolism, phagocytosis, and tissue repair. In parallel, downstream targets such as Cd36 and Arg1 are closely linked to reparative macrophage phenotypes and metabolic adaptation during tissue injury. Alterations in these pathways therefore suggest that ageing not only influences inflammatory activation but also modifies the capacity of pulmonary macrophages to initiate resolution programs following acute inflammatory injury [31, 32, 67–69].

The differential regulation of *Nos2*, *Hmox1*, and *Klotho* further emphasizes that oxidative stress and cytoprotective signaling represent integral components of age-dependent inflammatory responses. While inducible nitric oxide synthase contributes to antimicrobial defense, excessive nitric oxide production may aggravate oxidative tissue injury during acute inflammation. In contrast, heme oxygenase-1 is widely recognized as a stress-inducible cytoprotective enzyme that limits oxidative injury and modulates macrophage polarization. Likewise, Klotho has emerged as an important regulator of ageing-associated tissue homeostasis and resistance to oxidative stress. Together, these findings suggest that age-dependent pulmonary inflammation reflects not only altered inflammatory signaling but also changes in endogenous protective mechanisms that normally restrict tissue injury and facilitate recovery [33, 34, 45, 70].

Collectively, the transcriptional analyses extend the flow-cytometric findings by demonstrating that age-dependent immune remodeling is accompanied by coordinated changes in inflammatory, metabolic, and cytoprotective gene networks. Rather than supporting the concept of isolated cytokine dysregulation, our results indicate comprehensive reorganization of pulmonary transcriptional programs during acute lung injury, providing a mechanistic framework for the proteomic alterations discussed below.

While transcriptional analyses provide insight into inflammatory regulation, the proteomic data considerably extend these findings by demonstrating that biological ageing reshapes pulmonary inflammation at the functional protein level. In contrast to candidate-based gene expression analyses, the unbiased LC–MS/MS approach captures coordinated alterations across multiple biological pathways and therefore provides a more comprehensive view of the molecular processes governing acute lung injury. Our proteomic analyses revealed that ageing affects not only inflammatory signaling but also vascular integrity, extracellular matrix remodeling, hormone metabolism, and cytoprotective pathways, illustrating the complex biological remodeling that accompanies pulmonary inflammation during ageing [9, 11, 38, 39, 71].

One of the most prominent observations was the coordinated regulation of proteins involved in inflammatory signaling. Rather than isolated alterations of individual mediators, several components of canonical inflammatory pathways, including IL-1 signaling, NF-κB-associated proteins, MAP kinase signaling, and leukocyte activation, were differentially regulated according to age and sex. These findings support the concept that pulmonary inflammation is orchestrated through interconnected signaling networks rather than single cytokines and further explain why individual inflammatory markers frequently fail to reflect the biological complexity of ARDS. Similar pathway-oriented inflammatory remodeling has recently been proposed as a defining characteristic of biologically distinct ARDS endotypes [9–11, 48–51, 64].

Beyond inflammatory signaling, our data indicate substantial age-dependent alterations in proteins regulating endothelial barrier function and tissue architecture. Maintenance of alveolar-capillary integrity is essential for preserving pulmonary gas exchange, whereas disruption of endothelial junctions and extracellular matrix remodeling represent central pathological events during ARDS. The observed regulation of proteins associated with endothelial junctions, vascular permeability, and extracellular matrix turnover therefore suggests that biological ageing influences not only inflammatory activation but also structural mechanisms governing tissue injury and repair. This interpretation is supported by recent studies demonstrating extensive remodeling of both the pulmonary extracellular matrix and endothelial glycocalyx during acute lung injury [38, 39].

Interestingly, the proteomic analyses also identified proteins involved in steroid hormone metabolism and inflammatory regulation. Although these pathways have traditionally received less attention in experimental ARDS, increasing evidence indicates that endocrine signaling substantially modulates pulmonary inflammation and contributes to biological differences between males and females. Consequently, the observed regulation of steroid-associated proteins may provide one mechanistic explanation for the pronounced sex-dependent inflammatory phenotypes identified throughout our study. Future investigations should therefore address the functional interaction between hormonal signaling and pulmonary immune regulation during ageing [35–43].

Another important aspect emerging from the proteomic analyses is the close relationship between inflammatory activation and oxidative stress. Several proteins identified in the present study are functionally linked to reactive oxygen species generation, antioxidant defense, and inflammatory tissue injury. This observation complements the transcriptional regulation of Hmox1 and supports the concept that oxidative stress represents a central pathogenic mechanism during acute lung injury. Rather than acting independently, inflammatory signaling and oxidative stress constitute mutually reinforcing biological processes that contribute to epithelial injury, endothelial dysfunction, and impaired inflammatory resolution [33, 34, 70].

Importantly, the proteomic data closely mirror the findings obtained by flow cytometry and transcriptional profiling. Across all three analytical platforms, ageing consistently influenced inflammatory regulation through coordinated pathway remodeling rather than isolated molecular alterations. This concordance substantially strengthens the biological interpretation of our findings and underscores the value of integrating complementary analytical technologies when investigating complex inflammatory diseases such as ARDS. Instead of identifying individual biomarker candidates, the present study defines biological response patterns that may prove more informative for future patient stratification and therapeutic targeting [9–11, 48–51, 57–59].

A central strength of the present study is the integration of three independent biological data layers into a unified multidimensional analysis. While flow cytometry characterized age- and sex-dependent alterations in immune-cell composition, transcriptional profiling identified coordinated inflammatory and regulatory gene programs, and unbiased LC–MS/MS proteomics captured functional changes at the protein level. Integrating these complementary datasets by Uniform Manifold Approximation and Projection (UMAP) enabled visualization of the overall biological relationships between experimental groups and demonstrated that pulmonary inflammatory responses are organized according to distinct multidimensional response patterns rather than isolated molecular changes [9–11, 46–51, 57–60].

Importantly, UMAP should not be interpreted as a statistical validation of biological endotypes but rather visualizes similarities among multidimensional biological response profiles. Nevertheless, the clear separation of experimental groups observed in the present study indicates that biological ageing and sex consistently influence multiple levels of pulmonary immune regulation. The concordance between cellular, transcriptional, and proteomic alterations substantially strengthens the biological interpretation of our findings and supports the concept that inflammatory heterogeneity during acute lung injury extends across several interconnected molecular layers rather than reflecting isolated changes in individual biomarkers [9–11, 46–51].

These observations are highly consistent with emerging concepts of precision medicine in ARDS. Increasing evidence suggests that successful patient stratification will require integration of multiple biological domains, including immune-cell composition, circulating biomarkers, transcriptomics, proteomics, metabolomics, imageing, and clinical parameters. Rather than searching for a single predictive biomarker, future precision-medicine approaches will likely rely on multidimensional biological signatures that more accurately reflect the complexity of disease mechanisms. Our experimental strategy mirrors this concept by integrating complementary analytical platforms and thereby provides a framework for future translational studies investigating age- and sex-specific inflammatory endotypes in acute lung injury [9–11, 48–51, 57–60].

Several limitations of the present study should be considered. First, inflammatory responses were analyzed at a single early time point following intratracheal LPS administration. Although this model reliably captures the acute inflammatory phase of lung injury, it does not allow assessment of subsequent resolution, tissue repair, or long-term remodeling. Second, the LPS model reproduces many hallmarks of inflammatory lung injury but cannot fully reflect the complexity and clinical heterogeneity of human ARDS, which frequently results from polymicrobial infection, viral pneumonia, aspiration, or trauma [54, 56, 61, 72].

Third, our flow-cytometric analyses were based on relative immune-cell frequencies rather than absolute cell numbers. Although this approach enabled robust comparison of compartment-specific inflammatory responses across multiple tissues and minimized inter-experimental variability, future studies combining absolute leukocyte quantification with functional immune-cell analyses will further improve mechanistic understanding. Likewise, the transcriptional and proteomic analyses identify biological associations but do not establish causal relationships between individual signaling pathways and disease progression. Functional validation of candidate pathways will therefore be required in future experimental studies.

Despite these limitations, the present study provides a comprehensive systems-level characterization of age- and sex-dependent pulmonary inflammation during experimental acute lung injury. The consistent findings obtained across three independent analytical platforms strongly support the robustness of the observed biological patterns and emphasize the importance of integrating complementary molecular technologies when studying complex inflammatory diseases.

From a translational perspective, our findings reinforce the concept that biological ageing and sex should be incorporated into both experimental design and future clinical studies of ARDS. Therapeutic strategies targeting inflammatory pathways are unlikely to be equally effective across all patient populations if age- and sex-dependent biological differences are ignored. Instead, individualized treatment approaches based on integrated molecular phenotyping may improve patient stratification and increase the likelihood of successful targeted interventions. As high-dimensional immune profiling becomes increasingly feasible in clinical practice, multidimensional datasets similar to those generated in the present study may contribute to the identification of clinically relevant inflammatory endotypes and facilitate the implementation of precision medicine in critical care [9–11, 48–51, 57–60].

## Conclusion

In conclusion, our integrated analyses demonstrate that biological ageing and sex profoundly reshape pulmonary inflammatory responses during experimental acute lung injury at the cellular, transcriptional, and proteomic levels. Rather than inducing a uniform inflammatory phenotype, ageing promotes coordinated remodeling of immune-cell organization, inflammatory signaling, and molecular pathway activity in a compartment-specific manner. Integration of these multidimensional datasets identified distinct biological response patterns that support current concepts of inflammatory heterogeneity in ARDS and underscore the importance of incorporating age and sex into future experimental and clinical investigations. Collectively, our findings provide a comprehensive molecular framework for understanding age-associated pulmonary inflammation and may contribute to the development of future precision-medicine strategies for acute respiratory distress syndrome [9–11, 48–51, 57–60].

## Methods

### Induction of ALI: mouse housing and surgical procedure

This study is based on the cohort of C57BL/6 J mice obtained from Janvier Labs (Le Genest-Saint-Isle, France) as described in our earlier publication [55]. Aged (15–18 months) and young (8–12 weeks) male and female mice were included [55]. Mouse housing conditions, the induction of ALI, via an intratracheal application (i.t.) of LPS (5 µg/g bw LPS (Escherichia coli strain (O111:B4); Sigma-Aldrich; #L2630) or ultrapure water (sham) and anaesthesia followed by mechanical ventilation were carried out as previously outlined in the 1-h MICU model of Crispens and colleagues. Briefly, mice were anesthetized by intraperitoneal (i.p.) administration of ketamine (120 μg/g body weight), midazolam (1.25 μg/g body weight), and fentanyl (0.25 μg/g body weight; 10 mL/kg). After tracheotomy, mice were mechanically ventilated for 1 h using a lung-protective ventilation strategy while physiological parameters were continuously monitored [55].

### Flow Cytometric Analysis

Lymphoid, myeloid, and regulatory immune cell populations were analyzed in blood (150 µL), bronchoalveolar lavage fluid (BALF), lung, spleen, and mediastinal lymph nodes by flow cytometry using a BD FACSCanto™ II Flow Cytometer (BD Biosciences, Heidelberg, Germany). BALF was collected via tracheal incision using a 1 ml syringe with PBS. Following centrifugation (3,000 × g for 5 min) the cell pellet was separated from the supernatant and further used for flow cytometry staining. Lung, spleen, and lymph node tissues were dissociated using the Multi Tissue Dissociation Kit 1 (Miltenyi Biotec, Bergisch Gladbach, Germany; Cat. No. 130–110–201) in combination with the gentleMACS™ Octo Dissociator with Heaters according to the manufacturer's instructions using the organ-specific programs (Table S1) to obtain single-cell suspensions. Erythrocytes were removed by 10 min of hypotonic lysis using Red Blood Cell Lysis Solution (Miltenyi Biotec; Cat. No. 130–094–183). Cells were incubated for 15 min with an Fc receptor blocking reagent prior to staining with fluorochrome-conjugated monoclonal antibodies directed against the indicated surface markers. Surface staining was performed for 20 min at 4 °C in the dark. For the regulatory T-cell panel, cells were subsequently fixed, permeabilized, and stained intracellularly for FoxP3 using the BD FoxP3/Transcription Factor Staining Buffer Set (BD Biosciences, Heidelberg, Germany) according to the manufacturer's instructions. Intracellular FoxP3 staining was performed for 30 min at 4 °C.

The identification and differentiation of the corresponding immune cell subpopulations are carried out with the antibody combinations listed in Table S2. Gating strategies for lymphoid and myeloid panels, as well as T_regs_, are provided in Fig. S2A-C. BD FACSDiva software and FlowJo (Version 10.8.1) were used to analyse the cell characteristics. Immune-cell populations were quantified according to the respective gating hierarchy. Granulocytes, NK1.1^+^ cells, CD3^+^ T cells, and CD19^+^ B cells were expressed as the percentage of CD45 + leukocytes, with the CD45^+^ population serving as the parent population (100%). Monocytes/macrophages and dendritic cells were quantified as the percentage of the F4/80^+^Ly6G^−^ parent population. CD4^+^ and CD8α^+^ T-cell frequencies were expressed as the percentage of CD3^+^ T cells, whereas regulatory T cells (Tregs) were quantified as the percentage of CD3ε^+^CD4^+^ T cells. Because different immune-cell subsets were quantified relative to their respective parent populations, the reported frequencies are not mutually exclusive and therefore do not sum to 100%.

### Reverse Transcriptional-Quantitative Polymerase Chain Reaction (RT-qPCR)

Total RNA was isolated from the harvested lungs with Trizol Reagent (Sigma-Aldrich; 102679967) and reverse transcribed into cDNA with reverse transcriptase (Sigma-Aldrich; 9068–38-6), nonamers (Sigma-Aldrich; 1003430367), dNTPs (New England BioLabs Inc.; N0447L) and RNA inhibitor (New England BioLabs Inc.; M0314L). RT-qPCR was performed with Sybr Select Mastermix, DEPC (Roth; T143.3) and 1:10 dilutions in the listed primer sequences (Table S3). As an endogenous control, β-actin was added to each batch. Reaction conditions for RT-qPCR for the Step One Plus qPCR System (Applied Biosystems, Foster City, CA) were as follows: 50 °C for 2 min (activation of uracil-N-glycosylase (UDG)), 95 °C for 10 min and 45 cycles of 95 °C for 15 s and 59 °C for 1 min. A melt curve analysis was performed by 59 °C for 30 s, 65 °C for 5 s and with a melting rate of 0.5 °C/sec to 95 °C for 10 s. The fold change (FC) of each mRNA expression was quantified via the comparative C_T_ (∆∆C_T_) method.

### Proteomics

For proteome profiling, 60–100 mg murine lung tissue samples were homogenized with ReadyPrep™ Mini Grinders (BIO-RAD) in 400 μl urea lysis buffer (8 M urea, 20 mM HEPES, pH 8.0, 1 mM sodium orthovanadate, 2.5 mM sodium pyrophosphate, 1 mM beta-glycerophosphate) and sonicated 3 × for 10 s with an amplitude of 50% (Sonopuls TS102, Bandelin). Protein concentrations of the lysates were determined using the 660 nm assay kit (Thermo Fisher Scientific) according to the manufacturer’s instructions. 150 µg of protein per sample were reduced with DTT (10 mM for 1 h at 37 °C), alkylated with iodoacetamide (25 mM for 15 min at 37 °C in the dark) and digested using Lys-C (Wako/Fujifilm) for 2 h at 37 °C in an enzyme-to-substrate ratio of 1:50 (w/w). After dilution with 20 mM HEPES (pH 8.0) to a concentration of 2 M urea, digestion was continued overnight with trypsin (Promega) at 37 °C and a 1:50 (w/w) enzyme-to-substrate ratio. The peptide mixtures were acidified, purified using C18 spin tips (Havard) and dried by vacuum centrifugation. Afterward, the peptide samples were dissolved in 50 mM TEAB and peptide concentrations were determined using a fluorometric peptide assay (Thermo Fisher Scientific). Tandem mass tag (TMT) labelling of each sample was carried out according to the manufacturer's instructions. Briefly, 10 μg of peptides per sample were adjusted to a volume of 25 μl with 50 mM TEAB and incubated with 0.1 mg of TMT labelling reagent for 1 h at room temperature. After quenching the labelling reactions with hydroxylamine at a final concentration of 0.1%, the individually labelled samples were combined in a multiplex sample, which also contained a reference sample consisting of equal peptide amounts from each sample in one TMT channel. The TMT sample was dried by vacuum centrifugation and reconstituted in 0.1% TFA for pre-fractionation using a high-pH C18 reversed-phase kit (Thermo Fisher Scientific). The resulting 8 fractions were dried by vacuum centrifugation and stored at − 20 °C until mass spectrometric analysis. After reconstitution, the peptide mixtures were analyzed on a Q Exactive HF Orbitrap mass spectrometer coupled online to an Ultimate 3000 RSLCnano HPLC system (Thermo Fisher Scientific). The LC-MSMS measurement and further raw data processing with MaxQuant were performed as described previously in Nicolas et al. [73].

### Analysis of z-score-normalized datasets

The analysis and the following graphical representation of z-scores were performed using R (Version 4.4.3) with RStudio (Version 2024.12.1). Z-score normalized data from flow cytometry, proteomics and gene analysis (qPCR) were imported and formatted as feature-by-sample matrices. Before downstream analysis, it was ensured that feature identifiers were unique.

Principal Component Analyses (PCA) were performed separately for each dataset to reduce dimensionality. PCA was applied to normalized z-score-based matrices. Sample metadata, including sex, age and treatment, were incorporated to visualize and perform groupwise comparisons. For each dataset, the variance explained by each PC was calculated, and the cumulative variance of the eight PCs was evaluated. Key contributors to PC1 and PC2 were identified based on the absolute values of the loadings (Fig. S11A-C) [74]. Group-specific differences were visualized using gradient-colored scatter plots (Fig. S11D-F, upper panels) and heatmaps of the z-scores (Fig. S11D-F, lower panels). PCA results were visualized using Biplots, showing the top 5 contributing features of PC1. The first three PCs (PC1-3) from each dataset, which explained more than 80% of the variance, were combined and z-score normalized with respect to the between-sample variance for further analysis. Uniform Manifold Approximation and Projection (UMAP) was applied to the combined and normalized PCA data to visualize sample similarity in two dimensions.

Packages: *readxl, dplyr, tidyr, uwot, ggplot2, ggrepel, pheatmap*

Data import, processing, and formatting were performed in R using the readxl, dplyr, and tidyr packages. PCA generation was performed using prcomp(), and biplot visualization was performed using factoextra. Heatmaps were generated using the package pheatmap. uwot was used to generate UMAP. Graphical representations, including PCA and UMAP plots, were created using ggplot2. Group information (sex, age, treatment) was visualized using colors and symbols, and individual labels were added using ggrepel.

### Statistical analysis

Flow cytometric data were analyzed in R (version 4.4.3) using linear mixed-effects models (lme4 package). Fixed effects included age, sex, treatment (LPS vs. sham), and tissue compartment, whereas mouse identity was included as a random intercept to account for repeated measurements obtained from multiple tissues of the same animal. Model assumptions were assessed by visual inspection of residual and Q–Q plots. Estimated treatment effects (LPS − sham) together with corresponding p-values were extracted using the emmeans package. To account for multiple testing across immune-cell populations, p-values were adjusted using the Benjamini–Hochberg procedure. Adjusted p-values < 0.05 were considered statistically significant. Estimated treatment effects are presented as bubble heatmaps in which bubble color represents the direction of the response, bubble size reflects the absolute effect size (|estimate|), and black outlines indicate statistically significant differences after multiple-testing correction.

For the qPCR data, the ROUT test was carried out to identify outliers. The adjusted data were further checked for normality and lognormality. As the data were not normally distributed, the Kruskal–Wallis test with post hoc Dunn’s correction was applied to control family-wise error rates in multiple comparisons. The LPS mice were normalized to the corresponding shams, whereby the shams were set to 1. Similarly, the basal values of the aged sham groups compared to the young shams (= 1) were calculated using a One-Sample Wilcoxon Signed Rank Test. All data were presented as boxplots with medians, IQR, Q1, Q3, minimum, and maximum data values. We compared only young vs aged mice of the same sex, young female vs young male mice, and aged female vs aged male mice. We did not check young female vs aged male or young male vs aged female animals.

Because flow cytometric data comprised repeated measurements from multiple tissue compartments obtained from the same animals, linear mixed-effects models were applied to account for within-animal correlations. In contrast, qPCR data represented independent endpoint measurements from a single tissue and were therefore analyzed using conventional univariate statistical tests.

Proteomic data processing was carried out using R Studio (version 2024.09.1). First, potential contaminants, hits to the decoy database and proteins identified solely with modified peptides were removed. To control for equal sample loading, intensities from each LC-MSMS run were normalized on the median of the summed-up intensities from each sample. Furthermore, reporter intensities from replicate measurements and different TMT experiments were normalized by scaling to the internal reference containing equal peptide amounts of each sample [71]. Finally, data were filtered for proteins found in at least 70% of the samples. The remaining missing values were replaced by random numbers drawn from a normal distribution with a width of 0.3 and a down shift of 1.8. P- and Padj-values were considered significant at three levels: < 0.05 (*), < 0.01 (**), and < 0.001 (***).

## Supplementary Information


Supplementary Material 1.


## Data Availability

The datasets used and/or analysed during the current study are available from the corresponding author upon reasonable request.
