# Supplementary material for: Age- and sex-dependent heterogeneity during LPS-induced murine acute lung injury
Source: Immun Ageing. 2026 Aug 26;23:30. doi: 10.1186/s12979-026-00592-6 (PMC13528014; doi:10.1186/s12979-026-00592-6)
Supplement: Supplementary file 1 [file 12979_2026_592_MOESM1_ESM.docx]

**Age- and sex-dependent heterogeneity during LPS-induced murine acute lung injury**

Chantal Crispens^1^, Annett Wilken-Schmitz^1^, Valentin Dern^1^, Abd Rahman Rahimi^1^, Enrico Madau^1^, Nadine Chiara Frigger^1^, Josefine Jakob^2, 3^, Björn Häupl^3, 4^, Thomas Oellerich^2, 3, 4^, Andreas Weigert^5,6^, Steven R. Talbot^7,8^, Kai Zacharowski^1^, Ulrike Heinicke^1^ & Andreas von Knethen^1^

^1^Goethe University Frankfurt, University Hospital, Department of Anesthesiology, Intensive Care Medicine and Pain Therapy, Theodor-Stern-Kai 7, 60590 Frankfurt am Main, Germany

^2^Frankfurt Cancer Institute, Theodor-Stern-Kai 7, 60590 Frankfurt, Germany

^3^ Goethe University Frankfurt, University Hospital, Department of Hematology and Oncology, Theodor-Stern-Kai 7, 60590 Frankfurt, Germany

^4^German Cancer Consortium (DKTK), a partnership between DKFZ and UCT Frankfurt-Marburg, Germany, Frankfurt am Main, Germany

^5^Goethe University Frankfurt, Faculty of Medicine, Institute of Biochemistry I, 60590 Frankfurt, Germany

^6^Department for Immunity of Inflammation, Mannheim Institute for Innate Immunoscience (MI3), Medical Faculty Mannheim, Heidelberg University, Mannheim, Germany

^7^Hannover Medical School, Institute for Laboratory Animal Science, Carl-Neuberg-Straße 1, 30625, Hannover, Germany

^8^Institute of Medical Statistics, RWTH Aachen University Hospital, Aachen, Germany

Correspondence:

Prof. Dr. Andreas von Knethen

Goethe University Frankfurt

University Hospital Frankfurt

Department of Anesthesiology, Intensive Care Medicine and Pain Therapy

Theodor-Stern-Kai 7

60590 Frankfurt am Main

Germany

Email: andreas.vonknethen@unimedizin-ffm.de

**Supplementary Material**

**Supplementary Tables**

**Table S1**

Multispectral antibody panels were used to differentiate myeloid, lymphoid, and regulatory T cells (T_regs_) derived from lungs, BALFs, blood, spleens, and lymph nodes of young (8-12 weeks) and aged (15-18 months) female and male mice.

**Table S2**

Programs of the gentleMACS Octo Dissociator with heater used for preparing single cell suspensions of murine lungs, spleens, and lymph nodes.

**Table S3**

Primer pairs used in this study for qPCR.

**Table S4**

**Number of mice included in the flow cytometric analyses for each tissue, age, sex, and treatment group.**

| **Tissue** | **YM Sham** | **YM LPS** | **AM Sham** | **AM LPS** | **YF Sham** | **YF LPS** | **AF Sham** | **AF LPS** |
| --- | --- | --- | --- | --- | --- | --- | --- | --- |
| **Lung** | 4 | 5 | 8 | 7 | 7 | 7 | 7 | 8 |
| **BALF** | 9 | 10 | 12 | 12 | 14 | 14 | 13 | 14 |
| **Blood** | 10 | 6 | 8 | 7 | 8 | 8 | 7 | 8 |
| **Mediastinal lymph node** | 10 | 11 | 13 | 12 | 14 | 14 | 13 | 14 |
| **Spleen** | 10 | 11 | 13 | 12 | 14 | 14 | 13 | 14 |

Numbers indicate the mice included in the flow cytometric analyses for each tissue and experimental group. Sample sizes differ between tissues because lungs from a subset of animals were allocated to histological analyses and were therefore unavailable for flow cytometric analyses. YM, young male; AM, aged male; YF, young female; AF, aged female.

**Table S5. Results of the omnibus Type III tests from the linear mixed-effects models for all immune cell populations.**

| **Immune cell population** | **Effect** | **F** | **Num. df** | **Den. df** | **BH-adjusted P value** |
| --- | --- | --- | --- | --- | --- |
| Granulocytes | Age | 2.28 | 1 | 92 | 0.174 |
|  | Sex | 5.37 | 1 | 92 | **0.036** |
|  | LPS | 2.36 | 1 | 92 | 0.171 |
|  | Tissue | 1395.65 | 4 | 277 | **< 0.001** |
|  | Age x LPS | 1.99 | 1 | 92 | 0.197 |
|  | Sex x LPS | 1.45 | 1 | 92 | 0.253 |
|  | Tissue x LPS | 1.52 | 4 | 277 | 0.226 |
|  | Age x Sex x LPS | 0.93 | 1 | 92 | 0.359 |
| Monocytes/Macrophages | Age | 171.13 | 1 | 92 | **< 0.001** |
|  | Sex | 9.66 | 1 | 92 | **0.005** |
|  | LPS | 8.13 | 1 | 92 | **0.009** |
|  | Tissue | 111.56 | 4 | 277 | **< 0.001** |
|  | Age x LPS | 16.47 | 1 | 92 | **< 0.001** |
|  | Sex x LPS | 5.31 | 1 | 92 | **0.036** |
|  | Tissue x LPS | 4.93 | 4 | 277 | **0.002** |
|  | Age x Sex x LPS | 2.57 | 1 | 92 | 0.155 |
| DCs | Age | 176.42 | 1 | 92 | **< 0.001** |
|  | Sex | 8.92 | 1 | 92 | **0.006** |
|  | LPS | 9.02 | 1 | 92 | **0.006** |
|  | Tissue | 110.45 | 4 | 277 | **< 0.001** |
|  | Age x LPS | 15.41 | 1 | 92 | **<0.001** |
|  | Sex x LPS | 4.66 | 1 | 92 | **0.049** |
|  | Tissue x LPS | 5.15 | 4 | 277 | **0.001** |
|  | Age x Sex x LPS | 3.19 | 1 | 92 | 0.109 |
| Treg (FoxP3) | Age | 33.50 | 1 | 94 | **< 0.001** |
|  | Sex | 22.98 | 1 | 94 | **< 0.001** |
|  | LPS | 7.77 | 1 | 94 | **0.013** |
|  | Tissue | 63.41 | 4 | 273 | **< 0.001** |
|  | Age x LPS | 0.95 | 1 | 94 | 0.394 |
|  | Sex x LPS | 5.47 | 1 | 94 | **0.034** |
|  | Tissue x LPS | 4.12 | 4 | 273 | **0.007** |
|  | Age x Sex x LPS | 4.13 | 1 | 94 | 0.065 |
| NK1.1 | Age | 12.99 | 1 | 93 | **0.001** |
|  | Sex | 1.43 | 1 | 93 | 0.323 |
|  | LPS | 0.83 | 1 | 93 | 0.449 |
|  | Tissue | 397.55 | 4 | 282 | **< 0.001** |
|  | Age x LPS | 0.17 | 1 | 93 | 0.753 |
|  | Sex x LPS | 7.44 | 1 | 93 | **0.015** |
|  | Tissue x LPS | 16.95 | 4 | 282 | **< 0.001** |
|  | Age x Sex x LPS | 13.23 | 1 | 93 | **<0.001** |
| CD19 | Age | 30.02 | 1 | 93 | **< 0.001** |
|  | Sex | 5.87 | 1 | 93 | **0.031** |
|  | LPS | 0.09 | 1 | 93 | 0.794 |
|  | Tissue | 139.97 | 4 | 282 | **< 0.001** |
|  | Age x LPS | 3.24 | 1 | 93 | 0.118 |
|  | Sex x LPS | 0.33 | 1 | 93 | 0.656 |
|  | Tissue x LPS | 1.06 | 4 | 282 | 0.458 |
|  | Age x Sex x LPS | 20.94 | 1 | 93 | **< 0.001** |
| CD3 | Age | 42.72 | 1 | 93 | **< 0.001** |
|  | Sex | 2.46 | 1 | 93 | 0.181 |
|  | LPS | 0.06 | 1 | 93 | 0.818 |
|  | Tissue | 116.39 | 4 | 282 | **< 0.001** |
|  | Age x LPS | 2.56 | 1 | 93 | 0.173 |
|  | Sex x LPS | 1.55 | 1 | 93 | 0.304 |
|  | Tissue x LPS | 2.21 | 4 | 282 | 0.108 |
|  | Age x Sex x LPS | 16.02 | 1 | 93 | **< 0.001** |
| CD4 | Age | 0.62 | 1 | 93 | 0.509 |
|  | Sex | 0.15 | 1 | 93 | 0.753 |
|  | LPS | 0.21 | 1 | 93 | 0.734 |
|  | Tissue | 36.40 | 4 | 282 | **< 0.001** |
|  | Age x LPS | 0.91 | 1 | 93 | 0.429 |
|  | Sex x LPS | 0.13 | 1 | 93 | 0.753 |
|  | Tissue x LPS | 5.38 | 4 | 282 | **<0.001** |
|  | Age x Sex x LPS | 19.18 | 1 | 93 | **< 0.001** |
| CD8 | Age | 0.62 | 1 | 93 | 0.509 |
|  | Sex | 0.15 | 1 | 93 | 0.753 |
|  | LPS | 0.21 | 1 | 93 | 0.734 |
|  | Tissue | 36.40 | 4 | 282 | **< 0.001** |
|  | Age x LPS | 0.91 | 1 | 93 | 0.429 |
|  | Sex x LPS | 0.13 | 1 | 93 | 0.753 |
|  | Tissue x LPS | 5.38 | 4 | 282 | **<0.001** |
|  | Age x Sex x LPS | 19.18 | 1 | 93 | **< 0.001** |

Fixed effects (Age, Sex, LPS, and Tissue) and their interactions were assessed using Type III F-tests within the linear mixed-effects models. P values were adjusted for multiple testing within each flow cytometry panel using the Benjamini–Hochberg procedure. Significant BH-adjusted P values (<0.05) are shown in bold.

**Table S6. Pairwise post hoc comparisons (LPS vs. sham) derived from the linear mixed-effects models.**

| **Immune cell population** | **Compartment** | **Age** | **Sex** | **Estimate** | **SE** | **BH-adjusted**  **P value** |
| --- | --- | --- | --- | --- | --- | --- |
| Granulocytes | Lung | aged | female | -0.37 | 0.53 | 0.733 |
| Granulocytes | Lung | young | female | 0.85 | 0.56 | 0.359 |
| Granulocytes | Lung | aged | male | 1.34 | 0.53 | 0.065 |
| Granulocytes | Lung | young | male | 0.58 | 0.63 | 0.672 |
| Granulocytes | BALF | aged | female | -0.95 | 0.87 | 0.584 |
| Granulocytes | BALF | young | female | 0.38 | 0.87 | 0.865 |
| Granulocytes | BALF | aged | male | 0.15 | 0.87 | 0.978 |
| Granulocytes | BALF | young | male | 3.15 | 1.02 | **0.020** |
| Granulocytes | Blood | aged | female | -1.26 | 0.33 | **0.003** |
| Granulocytes | Blood | young | female | 0.38 | 0.33 | 0.558 |
| Granulocytes | Blood | aged | male | 0.65 | 0.35 | 0.204 |
| Granulocytes | Blood | young | male | -0.40 | 0.37 | 0.584 |
| Granulocytes | Mediastinal lymph node | aged | female | 0.00 | 0.05 | 1.000 |
| Granulocytes | Mediastinal lymph node | young | female | 0.00 | 0.05 | 1.000 |
| Granulocytes | Mediastinal lymph node | aged | male | -0.14 | 0.05 | **0.044** |
| Granulocytes | Mediastinal lymph node | young | male | 0.00 | 0.05 | 1.000 |
| Granulocytes | Spleen | aged | female | 0.08 | 0.21 | 0.865 |
| Granulocytes | Spleen | young | female | -0.09 | 0.21 | 0.865 |
| Granulocytes | Spleen | aged | male | -0.16 | 0.22 | 0.726 |
| Granulocytes | Spleen | young | male | -0.18 | 0.24 | 0.726 |
| Monocytes/Macrophages | Lung | aged | female | -3.82 | 0.49 | **<0.001** |
| Monocytes/Macrophages | Lung | young | female | -0.12 | 0.51 | 0.951 |
| Monocytes/Macrophages | Lung | aged | male | 1.01 | 0.50 | 0.165 |
| Monocytes/Macrophages | Lung | young | male | -0.16 | 0.58 | 0.951 |
| Monocytes/Macrophages | BALF | aged | female | -1.32 | 1.00 | 0.437 |
| Monocytes/Macrophages | BALF | young | female | 0.07 | 1.00 | 0.997 |
| Monocytes/Macrophages | BALF | aged | male | -1.60 | 1.00 | 0.322 |
| Monocytes/Macrophages | BALF | young | male | 0.76 | 1.17 | 0.758 |
| Monocytes/Macrophages | Blood | aged | female | 0.35 | 0.45 | 0.726 |
| Monocytes/Macrophages | Blood | young | female | 0.35 | 0.45 | 0.726 |
| Monocytes/Macrophages | Blood | aged | male | -1.31 | 0.48 | **0.044** |
| Monocytes/Macrophages | Blood | young | male | 1.81 | 0.52 | **0.006** |
| Monocytes/Macrophages | Mediastinal lymph node | aged | female | -1.41 | 0.57 | 0.065 |
| Monocytes/Macrophages | Mediastinal lymph node | young | female | 0.24 | 0.57 | 0.865 |
| Monocytes/Macrophages | Mediastinal lymph node | aged | male | -0.80 | 0.59 | 0.437 |
| Monocytes/Macrophages | Mediastinal lymph node | young | male | -1.28 | 0.64 | 0.169 |
| Monocytes/Macrophages | Spleen | aged | female | -2.20 | 0.41 | **<0.001** |
| Monocytes/Macrophages | Spleen | young | female | -0.05 | 0.42 | 0.978 |
| Monocytes/Macrophages | Spleen | aged | male | -0.44 | 0.42 | 0.584 |
| Monocytes/Macrophages | Spleen | young | male | 0.18 | 0.46 | 0.865 |
| DCs | Lung | aged | female | 3.82 | 0.49 | **<0.001** |
| DCs | Lung | young | female | 0.12 | 0.51 | 0.951 |
| DCs | Lung | aged | male | -1.01 | 0.50 | 0.165 |
| DCs | Lung | young | male | 0.27 | 0.57 | 0.865 |
| DCs | BALF | aged | female | 1.32 | 1.00 | 0.437 |
| DCs | BALF | young | female | -0.07 | 1.00 | 0.997 |
| DCs | BALF | aged | male | 1.60 | 1.00 | 0.322 |
| DCs | BALF | young | male | -0.82 | 1.17 | 0.733 |
| DCs | Blood | aged | female | -0.35 | 0.45 | 0.726 |
| DCs | Blood | young | female | -0.35 | 0.45 | 0.726 |
| DCs | Blood | aged | male | 1.31 | 0.48 | **0.044** |
| DCs | Blood | young | male | -1.81 | 0.52 | **0.006** |
| DCs | Mediastinal lymph node | aged | female | 1.41 | 0.57 | 0.065 |
| DCs | Mediastinal lymph node | young | female | -0.24 | 0.57 | 0.865 |
| DCs | Mediastinal lymph node | aged | male | 0.80 | 0.59 | 0.437 |
| DCs | Mediastinal lymph node | young | male | 1.28 | 0.64 | 0.169 |
| DCs | Spleen | aged | female | 2.20 | 0.41 | **<0.001** |
| DCs | Spleen | young | female | 0.05 | 0.41 | 0.978 |
| DCs | Spleen | aged | male | 0.44 | 0.42 | 0.584 |
| DCs | Spleen | young | male | 0.06 | 0.46 | 0.978 |
| FoxP3 | Lung | aged | female | -0.15 | 0.38 | 0.882 |
| FoxP3 | Lung | young | female | 0.15 | 0.29 | 0.882 |
| FoxP3 | Lung | aged | male | 0.05 | 0.30 | 0.908 |
| FoxP3 | Lung | young | male | 0.83 | 0.35 | 0.152 |
| FoxP3 | BALF | aged | female | -0.27 | 0.71 | 0.882 |
| FoxP3 | BALF | young | female | 0.97 | 0.71 | 0.578 |
| FoxP3 | BALF | aged | male | 0.25 | 0.71 | 0.882 |
| FoxP3 | BALF | young | male | -0.21 | 0.91 | 0.906 |
| FoxP3 | Blood | aged | female | 0.22 | 0.24 | 0.781 |
| FoxP3 | Blood | young | female | 0.10 | 0.23 | 0.882 |
| FoxP3 | Blood | aged | male | -0.04 | 0.25 | 0.908 |
| FoxP3 | Blood | young | male | -0.54 | 0.28 | 0.305 |
| FoxP3 | Mediastinal lymph node | aged | female | 0.95 | 0.21 | **<0.001** |
| FoxP3 | Mediastinal lymph node | young | female | 0.34 | 0.21 | 0.483 |
| FoxP3 | Mediastinal lymph node | aged | male | -0.06 | 0.22 | 0.882 |
| FoxP3 | Mediastinal lymph node | young | male | 0.63 | 0.24 | 0.100 |
| FoxP3 | Spleen | aged | female | 1.03 | 0.21 | **<0.001** |
| FoxP3 | Spleen | young | female | 0.11 | 0.20 | 0.882 |
| FoxP3 | Spleen | aged | male | -0.09 | 0.22 | 0.882 |
| FoxP3 | Spleen | young | male | -0.21 | 0.24 | 0.781 |
| NK1.1 | Lung | aged | female | -0.06 | 0.22 | 0.862 |
| NK1.1 | Lung | young | female | -0.08 | 0.21 | 0.838 |
| NK1.1 | Lung | aged | male | 0.07 | 0.22 | 0.858 |
| NK1.1 | Lung | young | male | 2.96 | 0.22 | **<0.001** |
| NK1.1 | BALF | aged | female | -0.97 | 0.53 | 0.229 |
| NK1.1 | BALF | young | female | -1.59 | 0.54 | **0.025** |
| NK1.1 | BALF | aged | male | 0.24 | 0.53 | 0.826 |
| NK1.1 | BALF | young | male | 0.69 | 0.68 | 0.592 |
| NK1.1 | Blood | aged | female | 0.25 | 0.22 | 0.567 |
| NK1.1 | Blood | young | female | -0.83 | 0.22 | **0.003** |
| NK1.1 | Blood | aged | male | 0.16 | 0.23 | 0.694 |
| NK1.1 | Blood | young | male | -0.64 | 0.26 | 0.082 |
| NK1.1 | Mediastinal lymph node | aged | female | 0.13 | 0.13 | 0.592 |
| NK1.1 | Mediastinal lymph node | young | female | -0.30 | 0.12 | 0.083 |
| NK1.1 | Mediastinal lymph node | aged | male | -0.15 | 0.13 | 0.567 |
| NK1.1 | Mediastinal lymph node | young | male | -0.29 | 0.14 | 0.157 |
| NK1.1 | Spleen | aged | female | -0.10 | 0.26 | 0.838 |
| NK1.1 | Spleen | young | female | -0.28 | 0.26 | 0.590 |
| NK1.1 | Spleen | aged | male | 0.00 | 0.27 | 0.990 |
| NK1.1 | Spleen | young | male | -0.22 | 0.30 | 0.694 |
| CD19 | Lung | aged | female | 0.25 | 0.33 | 0.694 |
| CD19 | Lung | young | female | 0.45 | 0.32 | 0.455 |
| CD19 | Lung | aged | male | 0.69 | 0.33 | 0.157 |
| CD19 | Lung | young | male | -1.11 | 0.33 | **0.010** |
| CD19 | BALF | aged | female | 1.69 | 0.83 | 0.157 |
| CD19 | BALF | young | female | -1.56 | 0.86 | 0.239 |
| CD19 | BALF | aged | male | 0.45 | 0.83 | 0.765 |
| CD19 | BALF | young | male | -2.83 | 1.08 | 0.057 |
| CD19 | Blood | aged | female | -0.67 | 0.20 | **0.011** |
| CD19 | Blood | young | female | 0.18 | 0.20 | 0.668 |
| CD19 | Blood | aged | male | 0.14 | 0.22 | 0.700 |
| CD19 | Blood | young | male | -0.35 | 0.24 | 0.442 |
| CD19 | Mediastinal lymph node | aged | female | -0.68 | 0.17 | **0.002** |
| CD19 | Mediastinal lymph node | young | female | 0.56 | 0.16 | **0.009** |
| CD19 | Mediastinal lymph node | aged | male | 0.19 | 0.17 | 0.590 |
| CD19 | Mediastinal lymph node | young | male | -0.06 | 0.19 | 0.858 |
| CD19 | Spleen | aged | female | -0.13 | 0.17 | 0.694 |
| CD19 | Spleen | young | female | 0.18 | 0.17 | 0.590 |
| CD19 | Spleen | aged | male | 0.08 | 0.18 | 0.827 |
| CD19 | Spleen | young | male | 0.22 | 0.20 | 0.567 |
| CD3 | Lung | aged | female | -0.24 | 0.34 | 0.694 |
| CD3 | Lung | young | female | -0.49 | 0.33 | 0.430 |
| CD3 | Lung | aged | male | -0.61 | 0.34 | 0.242 |
| CD3 | Lung | young | male | -0.03 | 0.34 | 0.979 |
| CD3 | BALF | aged | female | -1.64 | 0.64 | 0.064 |
| CD3 | BALF | young | female | 1.99 | 0.66 | **0.022** |
| CD3 | BALF | aged | male | -0.45 | 0.64 | 0.694 |
| CD3 | BALF | young | male | 1.11 | 0.83 | 0.476 |
| CD3 | Blood | aged | female | 0.65 | 0.20 | **0.013** |
| CD3 | Blood | young | female | -0.08 | 0.20 | 0.838 |
| CD3 | Blood | aged | male | -0.21 | 0.22 | 0.592 |
| CD3 | Blood | young | male | 0.49 | 0.24 | 0.157 |
| CD3 | Mediastinal lymph node | aged | female | 0.66 | 0.17 | **0.002** |
| CD3 | Mediastinal lymph node | young | female | -0.53 | 0.17 | **0.013** |
| CD3 | Mediastinal lymph node | aged | male | -0.24 | 0.18 | 0.474 |
| CD3 | Mediastinal lymph node | young | male | 0.05 | 0.19 | 0.862 |
| CD3 | Spleen | aged | female | 0.18 | 0.22 | 0.694 |
| CD3 | Spleen | young | female | -0.12 | 0.22 | 0.766 |
| CD3 | Spleen | aged | male | -0.08 | 0.23 | 0.838 |
| CD3 | Spleen | young | male | -0.20 | 0.25 | 0.694 |
| CD4 | Lung | aged | female | 0.10 | 0.26 | 0.838 |
| CD4 | Lung | young | female | -0.07 | 0.25 | 0.862 |
| CD4 | Lung | aged | male | 0.20 | 0.26 | 0.694 |
| CD4 | Lung | young | male | 0.00 | 0.26 | 0.990 |
| CD4 | BALF | aged | female | 0.01 | 0.86 | 0.990 |
| CD4 | BALF | young | female | -0.60 | 0.89 | 0.694 |
| CD4 | BALF | aged | male | -0.13 | 0.86 | 0.934 |
| CD4 | BALF | young | male | 1.29 | 1.11 | 0.567 |
| CD4 | Blood | aged | female | -0.52 | 0.12 | **0.002** |
| CD4 | Blood | young | female | 0.17 | 0.12 | 0.442 |
| CD4 | Blood | aged | male | -0.08 | 0.13 | 0.745 |
| CD4 | Blood | young | male | -0.59 | 0.15 | **0.002** |
| CD4 | Mediastinal lymph node | aged | female | -0.07 | 0.08 | 0.681 |
| CD4 | Mediastinal lymph node | young | female | 0.10 | 0.08 | 0.510 |
| CD4 | Mediastinal lymph node | aged | male | 0.31 | 0.09 | **0.005** |
| CD4 | Mediastinal lymph node | young | male | -0.09 | 0.09 | 0.592 |
| CD4 | Spleen | aged | female | 0.09 | 0.13 | 0.694 |
| CD4 | Spleen | young | female | 0.14 | 0.13 | 0.590 |
| CD4 | Spleen | aged | male | 0.29 | 0.14 | 0.157 |
| CD4 | Spleen | young | male | -0.31 | 0.15 | 0.157 |
| CD8 | Lung | aged | female | -0.10 | 0.26 | 0.838 |
| CD8 | Lung | young | female | 0.07 | 0.25 | 0.862 |
| CD8 | Lung | aged | male | -0.20 | 0.26 | 0.694 |
| CD8 | Lung | young | male | 0.00 | 0.26 | 0.990 |
| CD8 | BALF | aged | female | -0.01 | 0.86 | 0.990 |
| CD8 | BALF | young | female | 0.60 | 0.89 | 0.694 |
| CD8 | BALF | aged | male | 0.14 | 0.86 | 0.934 |
| CD8 | BALF | young | male | -1.29 | 1.12 | 0.567 |
| CD8 | Blood | aged | female | 0.52 | 0.12 | **0.002** |
| CD8 | Blood | young | female | -0.17 | 0.12 | 0.442 |
| CD8 | Blood | aged | male | 0.08 | 0.13 | 0.745 |
| CD8 | Blood | young | male | 0.59 | 0.15 | **0.002** |
| CD8 | Mediastinal lymph node | aged | female | 0.07 | 0.08 | 0.681 |
| CD8 | Mediastinal lymph node | young | female | -0.10 | 0.08 | 0.510 |
| CD8 | Mediastinal lymph node | aged | male | -0.31 | 0.09 | **0.005** |
| CD8 | Mediastinal lymph node | young | male | 0.09 | 0.09 | 0.592 |
| CD8 | Spleen | aged | female | -0.09 | 0.13 | 0.694 |
| CD8 | Spleen | young | female | -0.14 | 0.13 | 0.590 |
| CD8 | Spleen | aged | male | -0.29 | 0.14 | 0.157 |
| CD8 | Spleen | young | male | 0.31 | 0.15 | 0.157 |

Pairwise comparisons of LPS- versus Sham-treated mice stratified by age, sex, and tissue compartment. Estimates represent the difference between LPS- and Sham-treated mice (LPS − Sham) derived from the linear mixed-effects model. Positive estimates indicate higher values in LPS-treated mice, whereas negative estimates indicate higher values in Sham-treated mice. P values were adjusted for multiple testing using the Benjamini–Hochberg procedure within each panel. Significant BH-adjusted P values (< 0.05) are shown in bold.

**Table S7. Pairwise comparisons of Aged versus Young mice stratified by sex, treatment, and tissue compartment.**

| **Panel** | **Population** | **Contrast** | **Sex** | **Treatment** | **Compartment** | **Estimate** | **Lower 95% CI** | **Upper 95% CI** | **P value** | **BH-adjusted P value** |
| --- | --- | --- | --- | --- | --- | --- | --- | --- | --- | --- |
| Myeloid | Granulocytes | Aged vs. Young | Female | LPS | Lung | -1.122 | -2.229 | -0.015 | 0.047 | 0.106 |
| Myeloid | Granulocytes | Aged vs. Young | Male | LPS | Lung | 0.862 | -0.279 | 2.002 | 0.137 | 0.242 |
| Myeloid | Granulocytes | Aged vs. Young | Female | Sham | Lung | 0.098 | -0.963 | 1.159 | 0.855 | 0.976 |
| Myeloid | Granulocytes | Aged vs. Young | Male | Sham | Lung | 0.102 | -1.066 | 1.271 | 0.862 | 0.976 |
| Myeloid | Granulocytes | Aged vs. Young | Female | LPS | BALF | -2.357 | -4.084 | -0.631 | 0.008 | **0.024** |
| Myeloid | Granulocytes | Aged vs. Young | Male | LPS | BALF | 0.656 | -1.200 | 2.512 | 0.484 | 0.676 |
| Myeloid | Granulocytes | Aged vs. Young | Female | Sham | BALF | -1.025 | -2.751 | 0.701 | 0.241 | 0.381 |
| Myeloid | Granulocytes | Aged vs. Young | Male | Sham | BALF | 3.653 | 1.752 | 5.555 | 0.000 | **0.001** |
| Myeloid | Granulocytes | Aged vs. Young | Female | LPS | Blood | -0.813 | -1.467 | -0.159 | 0.015 | **0.038** |
| Myeloid | Granulocytes | Aged vs. Young | Male | LPS | Blood | 0.908 | 0.184 | 1.632 | 0.015 | **0.038** |
| Myeloid | Granulocytes | Aged vs. Young | Female | Sham | Blood | 0.826 | 0.172 | 1.480 | 0.014 | **0.038** |
| Myeloid | Granulocytes | Aged vs. Young | Male | Sham | Blood | -0.146 | -0.860 | 0.568 | 0.685 | 0.832 |
| Myeloid | Granulocytes | Aged vs. Young | Female | LPS | Mediastinal lymph node | 0.000 | -0.096 | 0.096 | 1.000 | 1.000 |
| Myeloid | Granulocytes | Aged vs. Young | Male | LPS | Mediastinal lymph node | 0.000 | -0.104 | 0.104 | 1.000 | 1.000 |
| Myeloid | Granulocytes | Aged vs. Young | Female | Sham | Mediastinal lymph node | 0.000 | -0.096 | 0.096 | 1.000 | 1.000 |
| Myeloid | Granulocytes | Aged vs. Young | Male | Sham | Mediastinal lymph node | 0.138 | 0.033 | 0.242 | 0.010 | **0.030** |
| Myeloid | Granulocytes | Aged vs. Young | Female | LPS | Spleen | 0.283 | -0.140 | 0.706 | 0.188 | 0.305 |
| Myeloid | Granulocytes | Aged vs. Young | Male | LPS | Spleen | -0.223 | -0.671 | 0.226 | 0.327 | 0.468 |
| Myeloid | Granulocytes | Aged vs. Young | Female | Sham | Spleen | 0.106 | -0.309 | 0.520 | 0.614 | 0.828 |
| Myeloid | Granulocytes | Aged vs. Young | Male | Sham | Spleen | -0.241 | -0.694 | 0.211 | 0.292 | 0.447 |
| Myeloid | Monocytes/Macs | Aged vs. Young | Female | LPS | Lung | -3.187 | -4.204 | -2.170 | < 0.001 | **< 0.001** |
| Myeloid | Monocytes/Macs | Aged vs. Young | Male | LPS | Lung | 0.233 | -0.822 | 1.289 | 0.662 | 0.828 |
| Myeloid | Monocytes/Macs | Aged vs. Young | Female | Sham | Lung | 0.509 | -0.471 | 1.489 | 0.305 | 0.447 |
| Myeloid | Monocytes/Macs | Aged vs. Young | Male | Sham | Lung | -0.942 | -2.021 | 0.136 | 0.086 | 0.161 |
| Myeloid | Monocytes/Macs | Aged vs. Young | Female | LPS | BALF | -1.420 | -3.402 | 0.562 | 0.158 | 0.264 |
| Myeloid | Monocytes/Macs | Aged vs. Young | Male | LPS | BALF | -0.501 | -2.632 | 1.630 | 0.642 | 0.828 |
| Myeloid | Monocytes/Macs | Aged vs. Young | Female | Sham | BALF | -0.032 | -2.014 | 1.949 | 0.974 | 1.000 |
| Myeloid | Monocytes/Macs | Aged vs. Young | Male | Sham | BALF | 1.856 | -0.326 | 4.038 | 0.095 | 0.172 |
| Myeloid | Monocytes/Macs | Aged vs. Young | Female | LPS | Blood | -0.906 | -1.809 | -0.002 | 0.049 | 0.106 |
| Myeloid | Monocytes/Macs | Aged vs. Young | Male | LPS | Blood | -3.307 | -4.304 | -2.309 | < 0.001 | **< 0.001** |
| Myeloid | Monocytes/Macs | Aged vs. Young | Female | Sham | Blood | -0.898 | -1.801 | 0.006 | 0.051 | 0.106 |
| Myeloid | Monocytes/Macs | Aged vs. Young | Male | Sham | Blood | -0.187 | -1.174 | 0.799 | 0.707 | 0.832 |
| Myeloid | Monocytes/Macs | Aged vs. Young | Female | LPS | Mediastinal lymph node | -5.912 | -7.041 | -4.784 | < 0.001 | **< 0.001** |
| Myeloid | Monocytes/Macs | Aged vs. Young | Male | LPS | Mediastinal lymph node | -3.092 | -4.315 | -1.869 | < 0.001 | **< 0.001** |
| Myeloid | Monocytes/Macs | Aged vs. Young | Female | Sham | Mediastinal lymph node | -4.267 | -5.395 | -3.138 | < 0.001 | **< 0.001** |
| Myeloid | Monocytes/Macs | Aged vs. Young | Male | Sham | Mediastinal lymph node | -3.565 | -4.798 | -2.333 | < 0.001 | **< 0.001** |
| Myeloid | Monocytes/Macs | Aged vs. Young | Female | LPS | Spleen | -2.119 | -2.944 | -1.294 | < 0.001 | **< 0.001** |
| Myeloid | Monocytes/Macs | Aged vs. Young | Male | LPS | Spleen | -4.877 | -5.755 | -3.999 | < 0.001 | **< 0.001** |
| Myeloid | Monocytes/Macs | Aged vs. Young | Female | Sham | Spleen | 0.032 | -0.778 | 0.843 | 0.937 | 1.000 |
| Myeloid | Monocytes/Macs | Aged vs. Young | Male | Sham | Spleen | -4.252 | -5.137 | -3.367 | < 0.001 | **< 0.001** |
| Myeloid | DCs | Aged vs. Young | Female | LPS | Lung | 3.185 | 2.171 | 4.199 | < 0.001 | **< 0.001** |
| Myeloid | DCs | Aged vs. Young | Male | LPS | Lung | -0.232 | -1.285 | 0.820 | 0.662 | 0.828 |
| Myeloid | DCs | Aged vs. Young | Female | Sham | Lung | -0.508 | -1.486 | 0.469 | 0.304 | 0.447 |
| Myeloid | DCs | Aged vs. Young | Male | Sham | Lung | 1.053 | -0.023 | 2.128 | 0.055 | 0.110 |
| Myeloid | DCs | Aged vs. Young | Female | LPS | BALF | 1.418 | -0.563 | 3.398 | 0.159 | 0.264 |
| Myeloid | DCs | Aged vs. Young | Male | LPS | BALF | 0.502 | -1.628 | 2.632 | 0.641 | 0.828 |
| Myeloid | DCs | Aged vs. Young | Female | Sham | BALF | 0.033 | -1.947 | 2.013 | 0.974 | 1.000 |
| Myeloid | DCs | Aged vs. Young | Male | Sham | BALF | -1.922 | -4.103 | 0.259 | 0.083 | 0.161 |
| Myeloid | DCs | Aged vs. Young | Female | LPS | Blood | 0.906 | 0.003 | 1.808 | 0.049 | 0.106 |
| Myeloid | DCs | Aged vs. Young | Male | LPS | Blood | 3.306 | 2.310 | 4.303 | < 0.001 | **< 0.001** |
| Myeloid | DCs | Aged vs. Young | Female | Sham | Blood | 0.898 | -0.005 | 1.800 | 0.051 | 0.106 |
| Myeloid | DCs | Aged vs. Young | Male | Sham | Blood | 0.187 | -0.799 | 1.173 | 0.707 | 0.832 |
| Myeloid | DCs | Aged vs. Young | Female | LPS | Mediastinal lymph node | 5.912 | 4.786 | 7.039 | < 0.001 | **< 0.001** |
| Myeloid | DCs | Aged vs. Young | Male | LPS | Mediastinal lymph node | 3.092 | 1.871 | 4.312 | < 0.001 | **< 0.001** |
| Myeloid | DCs | Aged vs. Young | Female | Sham | Mediastinal lymph node | 4.267 | 3.141 | 5.393 | < 0.001 | **< 0.001** |
| Myeloid | DCs | Aged vs. Young | Male | Sham | Mediastinal lymph node | 3.565 | 2.336 | 4.795 | < 0.001 | **< 0.001** |
| Myeloid | DCs | Aged vs. Young | Female | LPS | Spleen | 2.119 | 1.299 | 2.939 | < 0.001 | **< 0.001** |
| Myeloid | DCs | Aged vs. Young | Male | LPS | Spleen | 4.877 | 4.004 | 5.750 | < 0.001 | **< 0.001** |
| Myeloid | DCs | Aged vs. Young | Female | Sham | Spleen | -0.032 | -0.838 | 0.773 | 0.937 | 1.000 |
| Myeloid | DCs | Aged vs. Young | Male | Sham | Spleen | 4.499 | 3.619 | 5.379 | < 0.001 | **< 0.001** |
| Lymphocyte | NK1.1 | Aged vs. Young | Female | LPS | Lung | -0.345 | -0.766 | 0.077 | 0.108 | 0.225 |
| Lymphocyte | NK1.1 | Aged vs. Young | Male | LPS | Lung | -0.636 | -1.105 | -0.167 | 0.008 | **0.027** |
| Lymphocyte | NK1.1 | Aged vs. Young | Female | Sham | Lung | -0.364 | -0.800 | 0.072 | 0.100 | 0.218 |
| Lymphocyte | NK1.1 | Aged vs. Young | Male | Sham | Lung | 2.255 | 1.852 | 2.659 | < 0.001 | **< 0.001** |
| Lymphocyte | NK1.1 | Aged vs. Young | Female | LPS | BALF | 0.461 | -0.587 | 1.509 | 0.384 | 0.574 |
| Lymphocyte | NK1.1 | Aged vs. Young | Male | LPS | BALF | -0.678 | -1.863 | 0.508 | 0.259 | 0.425 |
| Lymphocyte | NK1.1 | Aged vs. Young | Female | Sham | BALF | -0.160 | -1.243 | 0.922 | 0.769 | 0.827 |
| Lymphocyte | NK1.1 | Aged vs. Young | Male | Sham | BALF | -0.225 | -1.464 | 1.014 | 0.720 | 0.810 |
| Lymphocyte | NK1.1 | Aged vs. Young | Female | LPS | Blood | 0.905 | 0.474 | 1.335 | < 0.001 | **< 0.001** |
| Lymphocyte | NK1.1 | Aged vs. Young | Male | LPS | Blood | 0.080 | -0.408 | 0.568 | 0.745 | 0.810 |
| Lymphocyte | NK1.1 | Aged vs. Young | Female | Sham | Blood | -0.180 | -0.619 | 0.259 | 0.418 | 0.588 |
| Lymphocyte | NK1.1 | Aged vs. Young | Male | Sham | Blood | -0.719 | -1.221 | -0.217 | 0.005 | **0.019** |
| Lymphocyte | NK1.1 | Aged vs. Young | Female | LPS | Mediastinal lymph node | 0.552 | 0.308 | 0.796 | < 0.001 | **< 0.001** |
| Lymphocyte | NK1.1 | Aged vs. Young | Male | LPS | Mediastinal lymph node | 0.210 | -0.059 | 0.479 | 0.125 | 0.250 |
| Lymphocyte | NK1.1 | Aged vs. Young | Female | Sham | Mediastinal lymph node | 0.128 | -0.120 | 0.377 | 0.309 | 0.475 |
| Lymphocyte | NK1.1 | Aged vs. Young | Male | Sham | Mediastinal lymph node | 0.072 | -0.199 | 0.344 | 0.599 | 0.758 |
| Lymphocyte | NK1.1 | Aged vs. Young | Female | LPS | Spleen | 0.275 | -0.240 | 0.790 | 0.292 | 0.456 |
| Lymphocyte | NK1.1 | Aged vs. Young | Male | LPS | Spleen | -0.131 | -0.700 | 0.437 | 0.647 | 0.789 |
| Lymphocyte | NK1.1 | Aged vs. Young | Female | Sham | Spleen | 0.096 | -0.428 | 0.621 | 0.716 | 0.810 |
| Lymphocyte | NK1.1 | Aged vs. Young | Male | Sham | Spleen | -0.353 | -0.926 | 0.220 | 0.224 | 0.373 |
| Lymphocyte | CD19 | Aged vs. Young | Female | LPS | Lung | 0.879 | 0.242 | 1.516 | 0.007 | **0.024** |
| Lymphocyte | CD19 | Aged vs. Young | Male | LPS | Lung | 0.654 | -0.055 | 1.362 | 0.070 | 0.167 |
| Lymphocyte | CD19 | Aged vs. Young | Female | Sham | Lung | 1.071 | 0.412 | 1.730 | 0.002 | **0.006** |
| Lymphocyte | CD19 | Aged vs. Young | Male | Sham | Lung | -1.145 | -1.750 | -0.539 | < 0.001 | **0.001** |
| Lymphocyte | CD19 | Aged vs. Young | Female | LPS | BALF | 3.603 | 1.945 | 5.261 | < 0.001 | **< 0.001** |
| Lymphocyte | CD19 | Aged vs. Young | Male | LPS | BALF | 2.847 | 0.972 | 4.723 | 0.003 | **0.012** |
| Lymphocyte | CD19 | Aged vs. Young | Female | Sham | BALF | 0.351 | -1.361 | 2.063 | 0.685 | 0.810 |
| Lymphocyte | CD19 | Aged vs. Young | Male | Sham | BALF | -0.438 | -2.400 | 1.523 | 0.658 | 0.793 |
| Lymphocyte | CD19 | Aged vs. Young | Female | LPS | Blood | 0.172 | -0.225 | 0.568 | 0.392 | 0.577 |
| Lymphocyte | CD19 | Aged vs. Young | Male | LPS | Blood | 0.882 | 0.433 | 1.331 | < 0.001 | **0.001** |
| Lymphocyte | CD19 | Aged vs. Young | Female | Sham | Blood | 1.018 | 0.614 | 1.423 | 0.000 | **0.000** |
| Lymphocyte | CD19 | Aged vs. Young | Male | Sham | Blood | 0.394 | -0.069 | 0.856 | 0.094 | 0.210 |
| Lymphocyte | CD19 | Aged vs. Young | Female | LPS | Mediastinal lymph node | 0.295 | -0.033 | 0.622 | 0.077 | 0.180 |
| Lymphocyte | CD19 | Aged vs. Young | Male | LPS | Mediastinal lymph node | 0.161 | -0.201 | 0.522 | 0.380 | 0.574 |
| Lymphocyte | CD19 | Aged vs. Young | Female | Sham | Mediastinal lymph node | 1.530 | 1.197 | 1.864 | < 0.001 | **< 0.001** |
| Lymphocyte | CD19 | Aged vs. Young | Male | Sham | Mediastinal lymph node | -0.089 | -0.453 | 0.275 | 0.629 | 0.786 |
| Lymphocyte | CD19 | Aged vs. Young | Female | LPS | Spleen | -0.293 | -0.629 | 0.042 | 0.086 | 0.195 |
| Lymphocyte | CD19 | Aged vs. Young | Male | LPS | Spleen | -0.273 | -0.643 | 0.098 | 0.147 | 0.272 |
| Lymphocyte | CD19 | Aged vs. Young | Female | Sham | Spleen | 0.013 | -0.328 | 0.355 | 0.938 | 0.938 |
| Lymphocyte | CD19 | Aged vs. Young | Male | Sham | Spleen | -0.128 | -0.501 | 0.245 | 0.498 | 0.638 |
| Lymphocyte | CD3 | Aged vs. Young | Female | LPS | Lung | -0.415 | -1.067 | 0.237 | 0.209 | 0.363 |
| Lymphocyte | CD3 | Aged vs. Young | Male | LPS | Lung | -0.072 | -0.798 | 0.653 | 0.843 | 0.897 |
| Lymphocyte | CD3 | Aged vs. Young | Female | Sham | Lung | -0.666 | -1.340 | 0.009 | 0.053 | 0.129 |
| Lymphocyte | CD3 | Aged vs. Young | Male | Sham | Lung | 0.505 | -0.117 | 1.127 | 0.110 | 0.225 |
| Lymphocyte | CD3 | Aged vs. Young | Female | LPS | BALF | -3.407 | -4.683 | -2.130 | 0.000 | **0.000** |
| Lymphocyte | CD3 | Aged vs. Young | Male | LPS | BALF | -0.936 | -2.380 | 0.509 | 0.201 | 0.360 |
| Lymphocyte | CD3 | Aged vs. Young | Female | Sham | BALF | 0.224 | -1.094 | 1.542 | 0.737 | 0.810 |
| Lymphocyte | CD3 | Aged vs. Young | Male | Sham | BALF | 0.623 | -0.887 | 2.133 | 0.415 | 0.588 |
| Lymphocyte | CD3 | Aged vs. Young | Female | LPS | Blood | -0.330 | -0.729 | 0.068 | 0.103 | 0.220 |
| Lymphocyte | CD3 | Aged vs. Young | Male | LPS | Blood | -0.964 | -1.415 | -0.513 | < 0.001 | **< 0.001** |
| Lymphocyte | CD3 | Aged vs. Young | Female | Sham | Blood | -1.057 | -1.463 | -0.650 | < 0.001 | **< 0.001** |
| Lymphocyte | CD3 | Aged vs. Young | Male | Sham | Blood | -0.257 | -0.721 | 0.207 | 0.274 | 0.435 |
| Lymphocyte | CD3 | Aged vs. Young | Female | LPS | Mediastinal lymph node | -0.332 | -0.661 | -0.003 | 0.048 | 0.127 |
| Lymphocyte | CD3 | Aged vs. Young | Male | LPS | Mediastinal lymph node | -0.203 | -0.566 | 0.160 | 0.270 | 0.435 |
| Lymphocyte | CD3 | Aged vs. Young | Female | Sham | Mediastinal lymph node | -1.530 | -1.865 | -1.194 | 0.000 | **0.000** |
| Lymphocyte | CD3 | Aged vs. Young | Male | Sham | Mediastinal lymph node | 0.087 | -0.280 | 0.453 | 0.640 | 0.789 |
| Lymphocyte | CD3 | Aged vs. Young | Female | LPS | Spleen | -0.035 | -0.468 | 0.399 | 0.875 | 0.910 |
| Lymphocyte | CD3 | Aged vs. Young | Male | LPS | Spleen | 0.327 | -0.152 | 0.805 | 0.178 | 0.324 |
| Lymphocyte | CD3 | Aged vs. Young | Female | Sham | Spleen | -0.326 | -0.767 | 0.116 | 0.146 | 0.272 |
| Lymphocyte | CD3 | Aged vs. Young | Male | Sham | Spleen | 0.205 | -0.277 | 0.687 | 0.400 | 0.580 |
| Lymphocyte | CD4 | Aged vs. Young | Female | LPS | Lung | -0.022 | -0.521 | 0.476 | 0.929 | 0.938 |
| Lymphocyte | CD4 | Aged vs. Young | Male | LPS | Lung | -0.701 | -1.255 | -0.146 | 0.014 | **0.041** |
| Lymphocyte | CD4 | Aged vs. Young | Female | Sham | Lung | -0.189 | -0.704 | 0.327 | 0.469 | 0.624 |
| Lymphocyte | CD4 | Aged vs. Young | Male | Sham | Lung | -0.904 | -1.379 | -0.429 | < 0.001 | **0.001** |
| Lymphocyte | CD4 | Aged vs. Young | Female | LPS | BALF | 0.319 | -1.390 | 2.027 | 0.712 | 0.810 |
| Lymphocyte | CD4 | Aged vs. Young | Male | LPS | BALF | -0.145 | -2.077 | 1.788 | 0.882 | 0.910 |
| Lymphocyte | CD4 | Aged vs. Young | Female | Sham | BALF | -0.289 | -2.054 | 1.475 | 0.745 | 0.810 |
| Lymphocyte | CD4 | Aged vs. Young | Male | Sham | BALF | 1.273 | -0.748 | 3.294 | 0.214 | 0.363 |
| Lymphocyte | CD4 | Aged vs. Young | Female | LPS | Blood | -0.597 | -0.838 | -0.356 | < 0.001 | **< 0.001** |
| Lymphocyte | CD4 | Aged vs. Young | Male | LPS | Blood | -0.209 | -0.481 | 0.064 | 0.132 | 0.253 |
| Lymphocyte | CD4 | Aged vs. Young | Female | Sham | Blood | 0.094 | -0.152 | 0.340 | 0.449 | 0.615 |
| Lymphocyte | CD4 | Aged vs. Young | Male | Sham | Blood | -0.719 | -0.999 | -0.439 | 0.000 | **0.000** |
| Lymphocyte | CD4 | Aged vs. Young | Female | LPS | Mediastinal lymph node | 0.165 | 0.004 | 0.327 | 0.045 | 0.123 |
| Lymphocyte | CD4 | Aged vs. Young | Male | LPS | Mediastinal lymph node | 0.177 | -0.002 | 0.355 | 0.052 | 0.129 |
| Lymphocyte | CD4 | Aged vs. Young | Female | Sham | Mediastinal lymph node | 0.338 | 0.173 | 0.503 | 0.000 | **0.001** |
| Lymphocyte | CD4 | Aged vs. Young | Male | Sham | Mediastinal lymph node | -0.226 | -0.406 | -0.046 | 0.014 | **0.041** |
| Lymphocyte | CD4 | Aged vs. Young | Female | LPS | Spleen | 0.520 | 0.262 | 0.779 | < 0.001 | **0.001** |
| Lymphocyte | CD4 | Aged vs. Young | Male | LPS | Spleen | 0.102 | -0.184 | 0.388 | 0.480 | 0.624 |
| Lymphocyte | CD4 | Aged vs. Young | Female | Sham | Spleen | 0.567 | 0.304 | 0.831 | 0.000 | **0.000** |
| Lymphocyte | CD4 | Aged vs. Young | Male | Sham | Spleen | -0.496 | -0.784 | -0.208 | 0.001 | **0.004** |
| Lymphocyte | CD8 | Aged vs. Young | Female | LPS | BALF | -0.319 | -2.028 | 1.390 | 0.712 | 0.810 |
| Lymphocyte | CD8 | Aged vs. Young | Male | LPS | BALF | 0.151 | -1.782 | 2.084 | 0.877 | 0.910 |
| Lymphocyte | CD8 | Aged vs. Young | Female | Sham | BALF | 0.289 | -1.476 | 2.054 | 0.745 | 0.810 |
| Lymphocyte | CD8 | Aged vs. Young | Male | Sham | BALF | -1.273 | -3.295 | 0.749 | 0.214 | 0.363 |
| Lymphocyte | CD8 | Aged vs. Young | Female | LPS | Lung | 0.022 | -0.476 | 0.521 | 0.929 | 0.938 |
| Lymphocyte | CD8 | Aged vs. Young | Male | LPS | Lung | 0.701 | 0.146 | 1.255 | 0.014 | **0.041** |
| Lymphocyte | CD8 | Aged vs. Young | Female | Sham | Lung | 0.189 | -0.327 | 0.704 | 0.469 | 0.624 |
| Lymphocyte | CD8 | Aged vs. Young | Male | Sham | Lung | 0.904 | 0.429 | 1.379 | < 0.001 | **0.001** |
| Lymphocyte | CD8 | Aged vs. Young | Female | LPS | Blood | 0.597 | 0.356 | 0.838 | < 0.001 | **< 0.001** |
| Lymphocyte | CD8 | Aged vs. Young | Male | LPS | Blood | 0.209 | -0.064 | 0.481 | 0.132 | 0.253 |
| Lymphocyte | CD8 | Aged vs. Young | Female | Sham | Blood | -0.094 | -0.340 | 0.152 | 0.449 | 0.615 |
| Lymphocyte | CD8 | Aged vs. Young | Male | Sham | Blood | 0.719 | 0.439 | 0.999 | < 0.001 | **< 0.001** |
| Lymphocyte | CD8 | Aged vs. Young | Female | LPS | Mediastinal lymph node | -0.165 | -0.327 | -0.004 | 0.045 | 0.123 |
| Lymphocyte | CD8 | Aged vs. Young | Male | LPS | Mediastinal lymph node | -0.177 | -0.355 | 0.002 | 0.052 | 0.129 |
| Lymphocyte | CD8 | Aged vs. Young | Female | Sham | Mediastinal lymph node | -0.338 | -0.503 | -0.173 | 0.000 | **0.001** |
| Lymphocyte | CD8 | Aged vs. Young | Male | Sham | Mediastinal lymph node | 0.226 | 0.046 | 0.406 | 0.014 | **0.041** |
| Lymphocyte | CD8 | Aged vs. Young | Female | LPS | Spleen | -0.520 | -0.779 | -0.262 | 0.000 | **0.001** |
| Lymphocyte | CD8 | Aged vs. Young | Male | LPS | Spleen | -0.102 | -0.388 | 0.184 | 0.480 | 0.624 |
| Lymphocyte | CD8 | Aged vs. Young | Female | Sham | Spleen | -0.567 | -0.831 | -0.304 | < 0.001 | **< 0.001** |
| Lymphocyte | CD8 | Aged vs. Young | Male | Sham | Spleen | 0.496 | 0.208 | 0.784 | 0.001 | **0.004** |
| Treg | FoxP3 | Aged vs. Young | Female | LPS | Lung | 0.554 | -0.022 | 1.131 | 0.059 | 0.089 |
| Treg | FoxP3 | Aged vs. Young | Male | LPS | Lung | -1.375 | -2.016 | -0.733 | < 0.001 | **< 0.001** |
| Treg | FoxP3 | Aged vs. Young | Female | Sham | Lung | 0.853 | 0.106 | 1.600 | 0.026 | **0.047** |
| Treg | FoxP3 | Aged vs. Young | Male | Sham | Lung | -0.596 | -1.249 | 0.056 | 0.073 | 0.104 |
| Treg | FoxP3 | Aged vs. Young | Female | LPS | BALF | 1.346 | -0.057 | 2.748 | 0.060 | 0.089 |
| Treg | FoxP3 | Aged vs. Young | Male | LPS | BALF | -1.352 | -2.938 | 0.234 | 0.094 | 0.125 |
| Treg | FoxP3 | Aged vs. Young | Female | Sham | BALF | 2.583 | 1.180 | 3.987 | < 0.001 | **0.001** |
| Treg | FoxP3 | Aged vs. Young | Male | Sham | BALF | -1.818 | -3.474 | -0.163 | 0.032 | 0.055 |
| Treg | FoxP3 | Aged vs. Young | Female | LPS | Blood | 0.603 | 0.142 | 1.064 | 0.011 | **0.022** |
| Treg | FoxP3 | Aged vs. Young | Male | LPS | Blood | 0.469 | -0.050 | 0.988 | 0.076 | 0.105 |
| Treg | FoxP3 | Aged vs. Young | Female | Sham | Blood | 0.484 | 0.015 | 0.954 | 0.043 | 0.072 |
| Treg | FoxP3 | Aged vs. Young | Male | Sham | Blood | -0.029 | -0.551 | 0.492 | 0.912 | 0.912 |
| Treg | FoxP3 | Aged vs. Young | Female | LPS | Mediastinal lymph node | 1.354 | 0.938 | 1.770 | < 0.001 | **< 0.001** |
| Treg | FoxP3 | Aged vs. Young | Male | LPS | Mediastinal lymph node | 0.172 | -0.288 | 0.632 | 0.459 | 0.483 |
| Treg | FoxP3 | Aged vs. Young | Female | Sham | Mediastinal lymph node | 0.745 | 0.321 | 1.169 | 0.001 | **0.002** |
| Treg | FoxP3 | Aged vs. Young | Male | Sham | Mediastinal lymph node | 0.870 | 0.410 | 1.331 | 0.000 | **0.001** |
| Treg | FoxP3 | Aged vs. Young | Female | LPS | Spleen | 1.094 | 0.689 | 1.500 | < 0.001 | **< 0.001** |
| Treg | FoxP3 | Aged vs. Young | Male | LPS | Spleen | 0.547 | 0.082 | 1.013 | 0.022 | **0.041** |
| Treg | FoxP3 | Aged vs. Young | Female | Sham | Spleen | 0.176 | -0.237 | 0.590 | 0.400 | 0.457 |
| Treg | FoxP3 | Aged vs. Young | Male | Sham | Spleen | 0.427 | -0.013 | 0.867 | 0.057 | 0.089 |

Pairwise comparisons of aged versus young mice stratified by sex, treatment, and tissue compartment. Estimates represent the difference between aged and young mice (Aged − Young) derived from the linear mixed-effects model. Positive estimates indicate higher values in aged mice, whereas negative estimates indicate higher values in young mice. P values were adjusted for multiple testing using the Benjamini–Hochberg procedure within each panel. Significant BH-adjusted P values (< 0.05) are shown in bold.

**Table S8. Pairwise comparisons of Female versus Male mice stratified by age, treatment, and tissue compartment.**

| **Panel** | **Population** | **Contrast** | **Age** | **Treatment** | **Compartment** | **Estimate** | **Lower 95% CI** | **Upper 95% CI** | **P value** | **BH-adjusted P value** |
| --- | --- | --- | --- | --- | --- | --- | --- | --- | --- | --- |
| Myeloid | Granulocytes | Female vs. Male | Aged | LPS | Lung | -1.740 | -2.801 | -0.679 | 0.002 | **0.007** |
| Myeloid | Granulocytes | Female vs. Male | Young | LPS | Lung | 0.244 | -0.940 | 1.427 | 0.683 | 0.932 |
| Myeloid | Granulocytes | Female vs. Male | Aged | Sham | Lung | -0.032 | -1.093 | 1.029 | 0.953 | 1.000 |
| Myeloid | Granulocytes | Female vs. Male | Young | Sham | Lung | -0.027 | -1.196 | 1.141 | 0.963 | 1.000 |
| Myeloid | Granulocytes | Female vs. Male | Aged | LPS | BALF | -2.927 | -4.653 | -1.201 | 0.001 | **0.006** |
| Myeloid | Granulocytes | Female vs. Male | Young | LPS | BALF | 0.086 | -1.770 | 1.942 | 0.927 | 1.000 |
| Myeloid | Granulocytes | Female vs. Male | Aged | Sham | BALF | -1.824 | -3.551 | -0.098 | 0.039 | 0.105 |
| Myeloid | Granulocytes | Female vs. Male | Young | Sham | BALF | 2.854 | 0.952 | 4.755 | 0.004 | **0.015** |
| Myeloid | Granulocytes | Female vs. Male | Aged | LPS | Blood | -2.003 | -2.687 | -1.319 | < 0.001 | **< 0.001** |
| Myeloid | Granulocytes | Female vs. Male | Young | LPS | Blood | -0.282 | -0.978 | 0.414 | 0.423 | 0.705 |
| Myeloid | Granulocytes | Female vs. Male | Aged | Sham | Blood | -0.089 | -0.755 | 0.577 | 0.790 | 1.000 |
| Myeloid | Granulocytes | Female vs. Male | Young | Sham | Blood | -1.062 | -1.765 | -0.359 | 0.003 | **0.015** |
| Myeloid | Granulocytes | Female vs. Male | Aged | LPS | Mediastinal lymph node | 0.000 | -0.098 | 0.098 | 1.000 | 1.000 |
| Myeloid | Granulocytes | Female vs. Male | Young | LPS | Mediastinal lymph node | 0.000 | -0.102 | 0.102 | 1.000 | 1.000 |
| Myeloid | Granulocytes | Female vs. Male | Aged | Sham | Mediastinal lymph node | -0.138 | -0.235 | -0.040 | 0.006 | **0.023** |
| Myeloid | Granulocytes | Female vs. Male | Young | Sham | Mediastinal lymph node | 0.000 | -0.103 | 0.103 | 1.000 | 1.000 |
| Myeloid | Granulocytes | Female vs. Male | Aged | LPS | Spleen | 0.079 | -0.344 | 0.502 | 0.711 | 0.945 |
| Myeloid | Granulocytes | Female vs. Male | Young | LPS | Spleen | -0.426 | -0.875 | 0.023 | 0.063 | 0.163 |
| Myeloid | Granulocytes | Female vs. Male | Aged | Sham | Spleen | -0.166 | -0.588 | 0.256 | 0.436 | 0.708 |
| Myeloid | Granulocytes | Female vs. Male | Young | Sham | Spleen | -0.513 | -0.958 | -0.068 | 0.024 | 0.072 |
| Myeloid | Monocytes/Macs | Female vs. Male | Aged | LPS | Lung | -3.192 | -4.176 | -2.208 | < 0.001 | **< 0.001** |
| Myeloid | Monocytes/Macs | Female vs. Male | Young | LPS | Lung | 0.228 | -0.858 | 1.315 | 0.677 | 0.932 |
| Myeloid | Monocytes/Macs | Female vs. Male | Aged | Sham | Lung | 1.643 | 0.660 | 2.626 | 0.001 | **0.006** |
| Myeloid | Monocytes/Macs | Female vs. Male | Young | Sham | Lung | 0.192 | -0.884 | 1.268 | 0.724 | 0.945 |
| Myeloid | Monocytes/Macs | Female vs. Male | Aged | LPS | BALF | -0.145 | -2.128 | 1.838 | 0.885 | 1.000 |
| Myeloid | Monocytes/Macs | Female vs. Male | Young | LPS | BALF | 0.774 | -1.356 | 2.903 | 0.472 | 0.726 |
| Myeloid | Monocytes/Macs | Female vs. Male | Aged | Sham | BALF | -0.424 | -2.407 | 1.558 | 0.672 | 0.932 |
| Myeloid | Monocytes/Macs | Female vs. Male | Young | Sham | BALF | 1.464 | -0.717 | 3.645 | 0.186 | 0.348 |
| Myeloid | Monocytes/Macs | Female vs. Male | Aged | LPS | Blood | 1.717 | 0.775 | 2.659 | 0.000 | **0.003** |
| Myeloid | Monocytes/Macs | Female vs. Male | Young | LPS | Blood | -0.683 | -1.644 | 0.277 | 0.161 | 0.323 |
| Myeloid | Monocytes/Macs | Female vs. Male | Aged | Sham | Blood | 0.065 | -0.855 | 0.985 | 0.889 | 1.000 |
| Myeloid | Monocytes/Macs | Female vs. Male | Young | Sham | Blood | 0.775 | -0.196 | 1.747 | 0.116 | 0.279 |
| Myeloid | Monocytes/Macs | Female vs. Male | Aged | LPS | Mediastinal lymph node | -1.322 | -2.474 | -0.169 | 0.025 | 0.072 |
| Myeloid | Monocytes/Macs | Female vs. Male | Young | LPS | Mediastinal lymph node | 1.499 | 0.299 | 2.699 | 0.015 | 0.050 |
| Myeloid | Monocytes/Macs | Female vs. Male | Aged | Sham | Mediastinal lymph node | -0.718 | -1.867 | 0.431 | 0.218 | 0.384 |
| Myeloid | Monocytes/Macs | Female vs. Male | Young | Sham | Mediastinal lymph node | -0.017 | -1.230 | 1.196 | 0.978 | 1.000 |
| Myeloid | Monocytes/Macs | Female vs. Male | Aged | LPS | Spleen | 2.140 | 1.313 | 2.968 | < 0.001 | **< 0.001** |
| Myeloid | Monocytes/Macs | Female vs. Male | Young | LPS | Spleen | -0.618 | -1.493 | 0.258 | 0.164 | 0.323 |
| Myeloid | Monocytes/Macs | Female vs. Male | Aged | Sham | Spleen | 3.905 | 3.080 | 4.730 | 0.000 | **< 0.001** |
| Myeloid | Monocytes/Macs | Female vs. Male | Young | Sham | Spleen | -0.380 | -1.251 | 0.492 | 0.389 | 0.667 |
| Myeloid | DCs | Female vs. Male | Aged | LPS | Lung | 3.188 | 2.207 | 4.169 | < 0.001 | **< 0.001** |
| Myeloid | DCs | Female vs. Male | Young | LPS | Lung | -0.229 | -1.312 | 0.855 | 0.676 | 0.932 |
| Myeloid | DCs | Female vs. Male | Aged | Sham | Lung | -1.641 | -2.621 | -0.661 | 0.001 | **0.006** |
| Myeloid | DCs | Female vs. Male | Young | Sham | Lung | -0.080 | -1.153 | 0.993 | 0.882 | 1.000 |
| Myeloid | DCs | Female vs. Male | Aged | LPS | BALF | 0.141 | -1.841 | 2.123 | 0.888 | 1.000 |
| Myeloid | DCs | Female vs. Male | Young | LPS | BALF | -0.774 | -2.902 | 1.354 | 0.472 | 0.726 |
| Myeloid | DCs | Female vs. Male | Aged | Sham | BALF | 0.426 | -1.555 | 2.408 | 0.670 | 0.932 |
| Myeloid | DCs | Female vs. Male | Young | Sham | BALF | -1.529 | -3.708 | 0.651 | 0.167 | 0.323 |
| Myeloid | DCs | Female vs. Male | Aged | LPS | Blood | -1.717 | -2.658 | -0.776 | 0.000 | **0.003** |
| Myeloid | DCs | Female vs. Male | Young | LPS | Blood | 0.683 | -0.277 | 1.644 | 0.161 | 0.323 |
| Myeloid | DCs | Female vs. Male | Aged | Sham | Blood | -0.065 | -0.984 | 0.854 | 0.889 | 1.000 |
| Myeloid | DCs | Female vs. Male | Young | Sham | Blood | -0.775 | -1.746 | 0.195 | 0.116 | 0.279 |
| Myeloid | DCs | Female vs. Male | Aged | LPS | Mediastinal lymph node | 1.322 | 0.172 | 2.472 | 0.025 | 0.072 |
| Myeloid | DCs | Female vs. Male | Young | LPS | Mediastinal lymph node | -1.499 | -2.697 | -0.301 | 0.015 | 0.050 |
| Myeloid | DCs | Female vs. Male | Aged | Sham | Mediastinal lymph node | 0.718 | -0.429 | 1.865 | 0.217 | 0.384 |
| Myeloid | DCs | Female vs. Male | Young | Sham | Mediastinal lymph node | 0.017 | -1.194 | 1.227 | 0.978 | 1.000 |
| Myeloid | DCs | Female vs. Male | Aged | LPS | Spleen | -2.140 | -2.963 | -1.317 | 0.000 | **0.000** |
| Myeloid | DCs | Female vs. Male | Young | LPS | Spleen | 0.618 | -0.253 | 1.488 | 0.162 | 0.323 |
| Myeloid | DCs | Female vs. Male | Aged | Sham | Spleen | -3.905 | -4.725 | -3.085 | < 0.001 | **< 0.001** |
| Myeloid | DCs | Female vs. Male | Young | Sham | Spleen | 0.626 | -0.240 | 1.493 | 0.154 | 0.323 |
| Lymphocyte | NK1.1 | Female vs. Male | Aged | LPS | Lung | -0.104 | -0.540 | 0.333 | 0.638 | 0.800 |
| Lymphocyte | NK1.1 | Female vs. Male | Young | LPS | Lung | -0.395 | -0.850 | 0.060 | 0.088 | 0.239 |
| Lymphocyte | NK1.1 | Female vs. Male | Aged | Sham | Lung | 0.023 | -0.413 | 0.460 | 0.916 | 0.936 |
| Lymphocyte | NK1.1 | Female vs. Male | Young | Sham | Lung | 2.643 | 2.240 | 3.046 | 0.000 | **< 0.001** |
| Lymphocyte | NK1.1 | Female vs. Male | Aged | LPS | BALF | -1.709 | -2.758 | -0.661 | 0.002 | **0.008** |
| Lymphocyte | NK1.1 | Female vs. Male | Young | LPS | BALF | -2.849 | -4.034 | -1.663 | < 0.001 | **< 0.001** |
| Lymphocyte | NK1.1 | Female vs. Male | Aged | Sham | BALF | -0.499 | -1.547 | 0.550 | 0.347 | 0.580 |
| Lymphocyte | NK1.1 | Female vs. Male | Young | Sham | BALF | -0.563 | -1.831 | 0.705 | 0.380 | 0.613 |
| Lymphocyte | NK1.1 | Female vs. Male | Aged | LPS | Blood | 0.481 | 0.032 | 0.929 | 0.036 | 0.128 |
| Lymphocyte | NK1.1 | Female vs. Male | Young | LPS | Blood | -0.344 | -0.815 | 0.128 | 0.151 | 0.322 |
| Lymphocyte | NK1.1 | Female vs. Male | Aged | Sham | Blood | 0.387 | -0.069 | 0.843 | 0.096 | 0.250 |
| Lymphocyte | NK1.1 | Female vs. Male | Young | Sham | Blood | -0.152 | -0.639 | 0.334 | 0.536 | 0.765 |
| Lymphocyte | NK1.1 | Female vs. Male | Aged | LPS | Mediastinal lymph node | 0.208 | -0.046 | 0.462 | 0.107 | 0.262 |
| Lymphocyte | NK1.1 | Female vs. Male | Young | LPS | Mediastinal lymph node | -0.134 | -0.394 | 0.126 | 0.308 | 0.550 |
| Lymphocyte | NK1.1 | Female vs. Male | Aged | Sham | Mediastinal lymph node | -0.068 | -0.322 | 0.185 | 0.593 | 0.780 |
| Lymphocyte | NK1.1 | Female vs. Male | Young | Sham | Mediastinal lymph node | -0.124 | -0.392 | 0.143 | 0.357 | 0.586 |
| Lymphocyte | NK1.1 | Female vs. Male | Aged | LPS | Spleen | 0.266 | -0.269 | 0.802 | 0.326 | 0.572 |
| Lymphocyte | NK1.1 | Female vs. Male | Young | LPS | Spleen | -0.140 | -0.689 | 0.409 | 0.614 | 0.787 |
| Lymphocyte | NK1.1 | Female vs. Male | Aged | Sham | Spleen | 0.369 | -0.165 | 0.903 | 0.174 | 0.362 |
| Lymphocyte | NK1.1 | Female vs. Male | Young | Sham | Spleen | -0.081 | -0.645 | 0.483 | 0.777 | 0.867 |
| Lymphocyte | CD19 | Female vs. Male | Aged | LPS | Lung | 0.089 | -0.570 | 0.748 | 0.789 | 0.867 |
| Lymphocyte | CD19 | Female vs. Male | Young | LPS | Lung | -0.136 | -0.824 | 0.552 | 0.695 | 0.818 |
| Lymphocyte | CD19 | Female vs. Male | Aged | Sham | Lung | 0.529 | -0.131 | 1.188 | 0.115 | 0.262 |
| Lymphocyte | CD19 | Female vs. Male | Young | Sham | Lung | -1.687 | -2.293 | -1.082 | 0.000 | **< 0.001** |
| Lymphocyte | CD19 | Female vs. Male | Aged | LPS | BALF | 1.751 | 0.093 | 3.409 | 0.039 | 0.133 |
| Lymphocyte | CD19 | Female vs. Male | Young | LPS | BALF | 0.996 | -0.880 | 2.871 | 0.295 | 0.535 |
| Lymphocyte | CD19 | Female vs. Male | Aged | Sham | BALF | 0.509 | -1.149 | 2.167 | 0.543 | 0.765 |
| Lymphocyte | CD19 | Female vs. Male | Young | Sham | BALF | -0.280 | -2.287 | 1.727 | 0.782 | 0.867 |
| Lymphocyte | CD19 | Female vs. Male | Aged | LPS | Blood | -0.970 | -1.383 | -0.557 | < 0.001 | **< 0.001** |
| Lymphocyte | CD19 | Female vs. Male | Young | LPS | Blood | -0.260 | -0.694 | 0.174 | 0.237 | 0.465 |
| Lymphocyte | CD19 | Female vs. Male | Aged | Sham | Blood | -0.160 | -0.580 | 0.260 | 0.452 | 0.695 |
| Lymphocyte | CD19 | Female vs. Male | Young | Sham | Blood | -0.784 | -1.233 | -0.336 | 0.001 | **0.004** |
| Lymphocyte | CD19 | Female vs. Male | Aged | LPS | Mediastinal lymph node | 0.031 | -0.309 | 0.372 | 0.856 | 0.911 |
| Lymphocyte | CD19 | Female vs. Male | Young | LPS | Mediastinal lymph node | -0.103 | -0.452 | 0.246 | 0.560 | 0.769 |
| Lymphocyte | CD19 | Female vs. Male | Aged | Sham | Mediastinal lymph node | 0.897 | 0.557 | 1.236 | < 0.001 | **< 0.001** |
| Lymphocyte | CD19 | Female vs. Male | Young | Sham | Mediastinal lymph node | -0.723 | -1.081 | -0.364 | < 0.001 | **0.001** |
| Lymphocyte | CD19 | Female vs. Male | Aged | LPS | Spleen | -0.190 | -0.539 | 0.159 | 0.282 | 0.522 |
| Lymphocyte | CD19 | Female vs. Male | Young | LPS | Spleen | -0.170 | -0.527 | 0.188 | 0.348 | 0.580 |
| Lymphocyte | CD19 | Female vs. Male | Aged | Sham | Spleen | 0.018 | -0.330 | 0.366 | 0.917 | 0.936 |
| Lymphocyte | CD19 | Female vs. Male | Young | Sham | Spleen | -0.123 | -0.491 | 0.244 | 0.508 | 0.758 |
| Lymphocyte | CD3 | Female vs. Male | Aged | LPS | Lung | -0.125 | -0.800 | 0.550 | 0.713 | 0.830 |
| Lymphocyte | CD3 | Female vs. Male | Young | LPS | Lung | 0.218 | -0.486 | 0.922 | 0.541 | 0.765 |
| Lymphocyte | CD3 | Female vs. Male | Aged | Sham | Lung | -0.496 | -1.171 | 0.179 | 0.148 | 0.321 |
| Lymphocyte | CD3 | Female vs. Male | Young | Sham | Lung | 0.674 | 0.053 | 1.296 | 0.034 | 0.128 |
| Lymphocyte | CD3 | Female vs. Male | Aged | LPS | BALF | -1.189 | -2.466 | 0.088 | 0.068 | 0.226 |
| Lymphocyte | CD3 | Female vs. Male | Young | LPS | BALF | 1.283 | -0.161 | 2.726 | 0.081 | 0.238 |
| Lymphocyte | CD3 | Female vs. Male | Aged | Sham | BALF | 0.008 | -1.269 | 1.285 | 0.990 | 0.990 |
| Lymphocyte | CD3 | Female vs. Male | Young | Sham | BALF | 0.407 | -1.138 | 1.952 | 0.602 | 0.782 |
| Lymphocyte | CD3 | Female vs. Male | Aged | LPS | Blood | 0.954 | 0.539 | 1.369 | < 0.001 | **< 0.001** |
| Lymphocyte | CD3 | Female vs. Male | Young | LPS | Blood | 0.321 | -0.115 | 0.757 | 0.148 | 0.321 |
| Lymphocyte | CD3 | Female vs. Male | Aged | Sham | Blood | 0.091 | -0.331 | 0.513 | 0.669 | 0.800 |
| Lymphocyte | CD3 | Female vs. Male | Young | Sham | Blood | 0.891 | 0.441 | 1.341 | < 0.001 | **0.001** |
| Lymphocyte | CD3 | Female vs. Male | Aged | LPS | Mediastinal lymph node | 0.003 | -0.340 | 0.345 | 0.987 | 0.990 |
| Lymphocyte | CD3 | Female vs. Male | Young | LPS | Mediastinal lymph node | 0.132 | -0.219 | 0.482 | 0.458 | 0.695 |
| Lymphocyte | CD3 | Female vs. Male | Aged | Sham | Mediastinal lymph node | -0.898 | -1.240 | -0.557 | < 0.001 | **< 0.001** |
| Lymphocyte | CD3 | Female vs. Male | Young | Sham | Mediastinal lymph node | 0.718 | 0.357 | 1.078 | < 0.001 | **0.001** |
| Lymphocyte | CD3 | Female vs. Male | Aged | LPS | Spleen | -0.140 | -0.591 | 0.311 | 0.539 | 0.765 |
| Lymphocyte | CD3 | Female vs. Male | Young | LPS | Spleen | 0.221 | -0.241 | 0.683 | 0.344 | 0.580 |
| Lymphocyte | CD3 | Female vs. Male | Aged | Sham | Spleen | -0.399 | -0.848 | 0.051 | 0.081 | 0.238 |
| Lymphocyte | CD3 | Female vs. Male | Young | Sham | Spleen | 0.132 | -0.342 | 0.607 | 0.581 | 0.775 |
| Lymphocyte | CD4 | Female vs. Male | Aged | LPS | Lung | 0.202 | -0.314 | 0.718 | 0.439 | 0.686 |
| Lymphocyte | CD4 | Female vs. Male | Young | LPS | Lung | -0.477 | -1.015 | 0.061 | 0.082 | 0.238 |
| Lymphocyte | CD4 | Female vs. Male | Aged | Sham | Lung | 0.300 | -0.216 | 0.815 | 0.252 | 0.475 |
| Lymphocyte | CD4 | Female vs. Male | Young | Sham | Lung | -0.415 | -0.890 | 0.060 | 0.086 | 0.238 |
| Lymphocyte | CD4 | Female vs. Male | Aged | LPS | BALF | 0.237 | -1.471 | 1.946 | 0.783 | 0.867 |
| Lymphocyte | CD4 | Female vs. Male | Young | LPS | BALF | -0.226 | -2.159 | 1.706 | 0.817 | 0.878 |
| Lymphocyte | CD4 | Female vs. Male | Aged | Sham | BALF | 0.094 | -1.614 | 1.803 | 0.913 | 0.936 |
| Lymphocyte | CD4 | Female vs. Male | Young | Sham | BALF | 1.657 | -0.412 | 3.725 | 0.115 | 0.262 |
| Lymphocyte | CD4 | Female vs. Male | Aged | LPS | Blood | -0.168 | -0.419 | 0.082 | 0.186 | 0.372 |
| Lymphocyte | CD4 | Female vs. Male | Young | LPS | Blood | 0.220 | -0.043 | 0.483 | 0.100 | 0.250 |
| Lymphocyte | CD4 | Female vs. Male | Aged | Sham | Blood | 0.275 | 0.020 | 0.530 | 0.035 | 0.128 |
| Lymphocyte | CD4 | Female vs. Male | Young | Sham | Blood | -0.538 | -0.809 | -0.266 | < 0.001 | **0.001** |
| Lymphocyte | CD4 | Female vs. Male | Aged | LPS | Mediastinal lymph node | -0.048 | -0.217 | 0.120 | 0.569 | 0.769 |
| Lymphocyte | CD4 | Female vs. Male | Young | LPS | Mediastinal lymph node | -0.037 | -0.209 | 0.135 | 0.672 | 0.800 |
| Lymphocyte | CD4 | Female vs. Male | Aged | Sham | Mediastinal lymph node | 0.333 | 0.165 | 0.500 | 0.000 | **0.001** |
| Lymphocyte | CD4 | Female vs. Male | Young | Sham | Mediastinal lymph node | -0.232 | -0.409 | -0.055 | 0.011 | **0.045** |
| Lymphocyte | CD4 | Female vs. Male | Aged | LPS | Spleen | 0.357 | 0.088 | 0.626 | 0.010 | **0.045** |
| Lymphocyte | CD4 | Female vs. Male | Young | LPS | Spleen | -0.061 | -0.337 | 0.214 | 0.660 | 0.800 |
| Lymphocyte | CD4 | Female vs. Male | Aged | Sham | Spleen | 0.558 | 0.290 | 0.827 | < 0.001 | **0.001** |
| Lymphocyte | CD4 | Female vs. Male | Young | Sham | Spleen | -0.505 | -0.788 | -0.222 | 0.001 | **0.003** |
| Lymphocyte | CD8 | Female vs. Male | Aged | LPS | Lung | -0.202 | -0.718 | 0.314 | 0.439 | 0.686 |
| Lymphocyte | CD8 | Female vs. Male | Young | LPS | Lung | 0.477 | -0.061 | 1.015 | 0.082 | 0.238 |
| Lymphocyte | CD8 | Female vs. Male | Aged | Sham | Lung | -0.300 | -0.815 | 0.216 | 0.252 | 0.475 |
| Lymphocyte | CD8 | Female vs. Male | Young | Sham | Lung | 0.415 | -0.060 | 0.890 | 0.086 | 0.238 |
| Lymphocyte | CD8 | Female vs. Male | Aged | LPS | BALF | -0.244 | -1.953 | 1.466 | 0.778 | 0.867 |
| Lymphocyte | CD8 | Female vs. Male | Young | LPS | BALF | 0.226 | -1.707 | 2.159 | 0.817 | 0.878 |
| Lymphocyte | CD8 | Female vs. Male | Aged | Sham | BALF | -0.094 | -1.804 | 1.615 | 0.913 | 0.936 |
| Lymphocyte | CD8 | Female vs. Male | Young | Sham | BALF | -1.657 | -3.726 | 0.413 | 0.115 | 0.262 |
| Lymphocyte | CD8 | Female vs. Male | Aged | LPS | Blood | 0.168 | -0.082 | 0.419 | 0.186 | 0.372 |
| Lymphocyte | CD8 | Female vs. Male | Young | LPS | Blood | -0.220 | -0.483 | 0.043 | 0.100 | 0.250 |
| Lymphocyte | CD8 | Female vs. Male | Aged | Sham | Blood | -0.275 | -0.530 | -0.020 | 0.035 | 0.128 |
| Lymphocyte | CD8 | Female vs. Male | Young | Sham | Blood | 0.538 | 0.266 | 0.809 | 0.000 | **0.001** |
| Lymphocyte | CD8 | Female vs. Male | Aged | LPS | Mediastinal lymph node | 0.048 | -0.120 | 0.217 | 0.569 | 0.769 |
| Lymphocyte | CD8 | Female vs. Male | Young | LPS | Mediastinal lymph node | 0.037 | -0.135 | 0.209 | 0.672 | 0.800 |
| Lymphocyte | CD8 | Female vs. Male | Aged | Sham | Mediastinal lymph node | -0.333 | -0.500 | -0.165 | 0.000 | **0.001** |
| Lymphocyte | CD8 | Female vs. Male | Young | Sham | Mediastinal lymph node | 0.232 | 0.055 | 0.409 | 0.011 | **0.045** |
| Lymphocyte | CD8 | Female vs. Male | Aged | LPS | Spleen | -0.357 | -0.626 | -0.088 | 0.010 | **0.045** |
| Lymphocyte | CD8 | Female vs. Male | Young | LPS | Spleen | 0.061 | -0.214 | 0.337 | 0.660 | 0.800 |
| Lymphocyte | CD8 | Female vs. Male | Aged | Sham | Spleen | -0.558 | -0.827 | -0.290 | < 0.001 | **0.001** |
| Lymphocyte | CD8 | Female vs. Male | Young | Sham | Spleen | 0.505 | 0.222 | 0.788 | 0.001 | **0.003** |
| Treg | FoxP3 | Female vs. Male | Aged | LPS | Lung | 1.102 | 0.504 | 1.699 | < 0.001 | **0.004** |
| Treg | FoxP3 | Female vs. Male | Young | LPS | Lung | -0.827 | -1.449 | -0.205 | 0.010 | **0.030** |
| Treg | FoxP3 | Female vs. Male | Aged | Sham | Lung | 1.306 | 0.558 | 2.055 | 0.001 | **0.005** |
| Treg | FoxP3 | Female vs. Male | Young | Sham | Lung | -0.143 | -0.794 | 0.507 | 0.663 | 0.829 |
| Treg | FoxP3 | Female vs. Male | Aged | LPS | BALF | 1.805 | 0.400 | 3.209 | 0.012 | **0.033** |
| Treg | FoxP3 | Female vs. Male | Young | LPS | BALF | -0.893 | -2.477 | 0.691 | 0.266 | 0.462 |
| Treg | FoxP3 | Female vs. Male | Aged | Sham | BALF | 2.326 | 0.922 | 3.731 | 0.001 | **0.007** |
| Treg | FoxP3 | Female vs. Male | Young | Sham | BALF | -2.075 | -3.730 | -0.421 | 0.015 | **0.034** |
| Treg | FoxP3 | Female vs. Male | Aged | LPS | Blood | 0.828 | 0.348 | 1.307 | 0.001 | **0.005** |
| Treg | FoxP3 | Female vs. Male | Young | LPS | Blood | 0.693 | 0.192 | 1.195 | 0.007 | **0.026** |
| Treg | FoxP3 | Female vs. Male | Aged | Sham | Blood | 0.566 | 0.088 | 1.045 | 0.021 | **0.046** |
| Treg | FoxP3 | Female vs. Male | Young | Sham | Blood | 0.053 | -0.461 | 0.566 | 0.839 | 0.976 |
| Treg | FoxP3 | Female vs. Male | Aged | LPS | Mediastinal lymph node | 1.183 | 0.750 | 1.616 | 0.000 | **< 0.001** |
| Treg | FoxP3 | Female vs. Male | Young | LPS | Mediastinal lymph node | 0.001 | -0.443 | 0.445 | 0.996 | 0.996 |
| Treg | FoxP3 | Female vs. Male | Aged | Sham | Mediastinal lymph node | 0.170 | -0.262 | 0.602 | 0.437 | 0.647 |
| Treg | FoxP3 | Female vs. Male | Young | Sham | Mediastinal lymph node | 0.295 | -0.158 | 0.748 | 0.199 | 0.362 |
| Treg | FoxP3 | Female vs. Male | Aged | LPS | Spleen | 0.977 | 0.555 | 1.400 | < 0.001 | **< 0.001** |
| Treg | FoxP3 | Female vs. Male | Young | LPS | Spleen | 0.430 | -0.020 | 0.881 | 0.061 | 0.116 |
| Treg | FoxP3 | Female vs. Male | Aged | Sham | Spleen | -0.140 | -0.562 | 0.281 | 0.510 | 0.729 |
| Treg | FoxP3 | Female vs. Male | Young | Sham | Spleen | 0.110 | -0.322 | 0.543 | 0.614 | 0.792 |

Pairwise comparisons of female versus male mice stratified by age, treatment, and tissue compartment. Estimates represent the difference between female and male mice (Female − Male) derived from the linear mixed-effects model. Positive estimates indicate higher values in female mice, whereas negative estimates indicate higher values in male mice. P values were adjusted for multiple testing using the Benjamini–Hochberg procedure within each panel. Significant BH-adjusted P values (< 0.05) are shown in bold.

**Table S9**

Sex-specific analyses of the proteome

**Table S10**

Age-specific analyses of the proteome

**Table S11**

Treatment-specific analyses of the proteome

**Table S12**

z-scores of lung immune cell subpopulations used for PCA and UMAP analyses.

**Table S13**

z-scores of lung mRNA expression used for PCA and UMAP analyses.

**Table S14**

z-scores of lung protein expression used for PCA and UMAP analyses.

**Supplementary Figures**

**Fig. S1 | Study design.** Young (8–12 weeks) and aged (15–18 months) female and male C57BL/6J mice were treated intratracheally (IT) with lipopolysaccharide (LPS; 5 µg/g body weight) or ultrapure water (Sham). After 6 h, animals underwent 1 h of mechanical ventilation in the mouse intensive care unit (MICU). Subsequently, lung tissue, bronchoalveolar lavage fluid (BALF), blood, spleen, and mediastinal lymph nodes were harvested for flow cytometry. Lung tissue was additionally subjected to RT-qPCR and LC–MS/MS-based proteomic analysis.

**Fig. S2 | Gating strategies of immune cell subpopulations.** Gating of immune cells derived from lung, BALF, blood, spleen, and mediastinal lymph node, exemplified for a blood sample derived from a young sham-treated male mouse. (A) Cells were gated for a myeloid panel. First, cells were analyzed according to size using the forward scatter (FSC-A) and the granularity via the side scatter (SSC-A). Doublettes were discriminated with FSC-W vs. FSC-A. Then, the remaining cells were stained for CD45, and only CD45^+^ cells were further analyzed. Staining for Ly6G vs. F4/80 revealed Ly6G^+^/F4/80^-^ cells, which were further analyzed for Ly6C expression and absent CD11c expression, leading to a Ly6G^+^Ly6C^+^CD11c^-^ cell population, indicative for granulocytes (green box). Gating on the F4/80^+^Ly6G^-^ cells, further differentiated by Ly6C and CD11c staining identified monocytes/macrophages (F4/80^+^Ly6G^-^ CD11c^-^Ly6C^+^, red gate) and dendritic cells (F4/80^+^Ly6G^-^Ly6C^-^CD11c^+^, violet gate). (B) Similarly to (A), cells were first evaluated based on size (FSC-A) vs. granularity (SSC-A). Following discrimination of doublettes (FSC-W vs. FSC-A), the lymphoid cell population was examined for CD45 expression. Further analysis of CD45^+^ cells was done to differentiate NK cells by staining for NK1.1 (brown gate). NK1.1^-^ cells were differentiated into CD19^+^ B (yellow gate) and CD3e^+^ T (green gate) cells. The latter were further differentiated into CD4^+^ T helper cells (T_h_, red gate) and CD8^+^ cytotoxic T cells (CTL, violet gate). (C) To stain T_regs_, similarly to (A) and (B), cells were first evaluated based on size (FSC-A) vs. granularity (SSC-A). Following discrimination of doublettes (FSC-W vs. FSC-A), the lymphoid cell population was examined for CD3ε expression in combination with CD4 expression to identify T helper cells (black gate), which were finally stained for intracellular FoxP3 protein expression to identify T_regs_ (green gate).

**Fig. S3 | Fewer macrophages and no increase in granulocytes in the lung immune cell profile revealed an immunocompromised state in aged female mice in response to LPS.** Flow cytometric analyses of pulmonary myeloid immune cells derived from lungs of sham and LPS-stimulated young and aged female as well as male mice. Gating was performed as outlined in Fig. S2A. Single cell preparations of lungs were stained for granulocytes, characterized by surface expression of LyG6^+^, F4/80^-^, Ly6C^+^, CD11c^-^ in (A) LPS normalized to sham and (B) sham showing age-dependent significant increased granulocyte counts in young females compared to aged counterparts. Macrophages (MFs) distinguished by Ly6G^+^, F4/80^+^, Ly6C^-^, CD11c^+^ ((C) LPS normalized to sham, (D) sham), were significantly decreased in aged female LPS-treated mice. Dendritic cells (DCs) showing Ly6G^-^, F4/80^+^, Ly6C^+^ CD11c^-^ ((E) LPS normalized to sham, (F) sham) were significantly increased in aged female animals. Total CD45^+^ cells were defined as 100% for granulocytes (B), and total percentages of MFs and DCs were set as 100% to calculate their ratio ((D), (F)). Data are shown as whisker box plots providing medians, IQR, Q1, Q3, minimum, and maximum data values. Mice included in light red, young female; blazing red, aged female; light blue, young male; royal blue, aged male mice; dotted green line (= sham) was set as 1. Statistical inference was performed using linear mixed-effects models. Corresponding pairwise comparisons and BH-adjusted P values are provided in Supplementary Tables S5–S7. IT, intratracheal; LPS, lipopolysaccharide.

**Fig. S4 | Flow cytometric analyses uncovered sex- and age-dependent variations in BALF cells of the innate immunity.** Flow cytometric analyses of myeloid immune cells derived from BALF of sham and LPS-stimulated young and aged female and male mice. Gating was performed as outlined in Fig. S2A. Single cell preparations were stained for granulocytes, characterized by surface expression of LyG6^+^, F4/80^-^, Ly6C^+^, CD11c^-^ in (A) LPS normalized to sham and (B) sham, macrophages (MFs) distinguished by Ly6G^+^, F4/80^+^, Ly6C^-^, CD11c^+^ ((C) LPS normalized to sham, (D) sham), and dendritic cells (DCs) showing Ly6G^-^, F4/80^+^, Ly6C^+^ CD11c^-^ ((E) LPS normalized to sham, (F) sham). Total CD45^+^ cells were defined as 100% for granulocytes (B), and the total percentage of MFs and DCs was set as 100% to calculate their ratio ((D), (F)). Data are shown as whisker box plots providing medians, IQR, Q1, Q3, minimum, and maximum data values. Light red, young female; blazing red, aged female; light blue, young male; royal blue, aged male mice; dotted green line (= sham) was set as 1. Statistical inference was performed using linear mixed-effects models. Corresponding pairwise comparisons and BH-adjusted P values are provided in Supplementary Tables S5–S7. IT, intratracheal; LPS, lipopolysaccharide.

**Fig. S5 | Flow cytometric analyses uncovered sex- and age-dependent variations in lung cells of the adaptive immunity.** Flow cytometric analyses of lymphoid immune cells derived from lungs of sham and LPS-stimulated young and aged female and male mice. Gating was performed as outlined in Fig. S2B. Single cell preparations of lungs were stained for NK cells (NK1.1), characterized by surface expression of NK1.1 ((A), (B)), B cells distinguished by CD19^+^, NK1.1^-^ (C, D), and T cells showing CD3^+^, CD19^-^, NK1.1^-^ ((E), (F)). T helper (T_h_) cells were defined by expressing CD4^+^CD3^+^CD8^-^CD19^-^NK1.1^-^ ((G), (H)) and cytotoxic T cells by surface staining of CD8^+^CD3^+^CD4^-^CD19^-^NK1.1^-^ ((I), (J)). Total CD45^+^ cells were defined as 100% for NK cells (B), B cells (D), and T cells (F). CD4^+^ and CD8^+^ cells were determined considering all CD3^+^ T cells set as 100%((H), (J)). Data are shown as whisker box plots providing medians, IQR, Q1, Q3, minimum, and maximum data values. When samples were normalized ((A), (C), (E), (G), (I)), sham is set as 1. Statistical inference was performed using linear mixed-effects models. Results of the omnibus tests and corresponding pairwise comparisons with BH-adjusted P values are provided in Supplementary Tables S5–S8. Light red, young female; blazing red, aged female; light blue, young male; royal blue, aged male mice; dotted green line (= sham) was set as 1. IT, intratracheal; LPS, lipopolysaccharide; T_h_, T helper cells.

**Fig. S6 | Flow cytometric profiling of the lymphoid BALF immune cell subtype.** Flow cytometric analyses of lymphoid immune cells derived from BALFs of sham and LPS-stimulated young and aged female as well as male mice. Gating was performed as outlined in Fig. S2B. Single cell preparations were stained for NK cells (NK1.1), characterized by surface expression of NK1.1 ((A), (B)), B cells distinguished by CD19^+^, NK1.1^-^ ((C), (D)), and T cells showing CD3^+^, CD19^-^, NK1.1^-^ ((E), (F)). T helper (T_h_) cells were defined expressing CD4^+^CD3^+^CD8^-^CD19^-^NK1.1^-^ ((G), (H)) and cytotoxic T cells by surface staining of CD8^+^CD3^+^CD4^-^CD19^-^NK1.1^-^ ((I), (J)). Total CD45^+^ cells were defined as 100% for NK cells (B), B cells (D) and T cells (F). CD4^+^ and CD8^+^ cells were determined considering all CD3^+^ T cells set as 100% ((H), (J)). Data are shown as whisker box plots providing medians, IQR, Q1, Q3, minimum, and maximum data values. Statistical differences were assessed via the Kruskal-Wallis test for non-normally distributed data and the ordinary one-way ANOVA when normally distributed. When samples were normalized ((A), (C), (E), (G)), sham was set as 1. Light red, young female; blazing red, aged female; light blue, young male; royal blue, aged male mice; dotted green line (= sham) was set as 1. Statistical inference was performed using linear mixed-effects models. Results of the omnibus tests and corresponding pairwise comparisons with BH-adjusted P values are provided in Supplementary Tables S5–S8. BALF, bronchoalveolar lavage fluid; IT, intratracheal; LPS, lipopolysaccharide.

**Fig. S7 | Flow cytometric analyses of lung-derived regulatory T cells.** Flow cytometric analyses of pulmonary regulatory T cells (T_reg_) derived from lungs of sham and LPS-stimulated young and aged female as well as male mice. Gating was performed as outlined in Fig. S2C. Single-cell preparations of lungs were stained for T_regs_ characterized by surface expression of CD4 and intracellular expression of FoxP3 ((A), (B)). Total CD4^+^ T cells were set as 100% (B). Data are shown as whisker box plots providing medians, IQR, Q1, Q3, minimum, and maximum data values. When samples were normalized (A), sham is set as 1. Results of the omnibus tests and corresponding pairwise comparisons with BH-adjusted P values are provided in Supplementary Tables S5–S8. Light red, young female; blazing red, aged female; light blue, young male; royal blue, aged male mice; dotted green line (= sham) was set as 1. IT, intratracheal; LPS, lipopolysaccharide.

**Fig. S8 | Flow cytometric analyses of BALF-derived regulatory T cells.** Flow cytometric analyses of pulmonary regulatory T cells (T_reg_) derived from BALFs of sham and LPS-stimulated young and aged female as well as male mice. Gating was performed as outlined in Fig. S2C. Single cell preparations were stained for T_regs_ characterized by surface expression of CD4 and intracellular expression of FoxP3 ((A), (B)). Total CD4^+^ T cells were set as 100% (B). Data are shown as whisker box plots providing medians, IQR, Q1, Q3, minimum, and maximum data values. When samples were normalized (A), sham is set as 1. Statistical inference was performed using linear mixed-effects models. Results of the omnibus tests and corresponding pairwise comparisons with BH-adjusted P values are provided in Supplementary Tables S5–S8. Light red, young female; blazing red, aged female; light blue, young male; royal blue, aged male mice; dotted green line (= sham) was set as 1. BALF, broncho-alveolar lavage fluid; IT, intratracheal; LPS, lipopolysaccharide.

Fig. S9

**Figure S9 | Linear mixed-effects analysis identifies age- and sex-dependent immune signatures in blood, mediastinal lymph nodes, and spleen following intratracheal LPS administration.** Flow-cytometric immune-cell frequencies were analyzed using linear mixed-effects models including age, sex, treatment (LPS vs. sham), and tissue compartment as fixed effects and mouse as a random intercept. Estimated treatment effects (LPS − sham) are displayed as bubble heatmaps for blood, mediastinal lymph nodes, and spleen. Rows represent the analyzed immune-cell populations grouped into myeloid (granulocytes, monocytes/macrophages, dendritic cells), lymphoid (NK1.1⁺, CD3⁺, CD4⁺, CD8⁺, and CD19⁺ cells), and regulatory (FoxP3⁺ Tregs) compartments. Columns represent aged and young animals separated by sex. Bubble color indicates the estimated direction and magnitude of the LPS-induced response relative to the corresponding sham controls (red = higher after LPS; blue = lower after LPS). Bubble size reflects the absolute effect size (|estimate|). Bubbles outlined in black indicate statistically significant treatment effects after Benjamini–Hochberg correction (BH-adjusted *p* < 0.05). Immune-cell frequencies were determined by multicolor flow cytometry using the gating strategy shown in Supplementary Fig. S2 and were calculated relative to the respective parent populations as described in the Methods. MLN, mediastinal lymph node. The corresponding bar graphs displaying the underlying flow-cytometric frequencies for blood, mediastinal lymph nodes, and spleen are provided in Supplementary Figs. S10–S17.

**Fig. S10 | Flow cytometric profiling of the myeloid immune cell subtypes in the blood of young and aged sham mice of both sexes.** Flow cytometric analyses of myeloid immune cells derived from the blood of sham and LPS-treated young (8-12 weeks) and aged (15 -18 months) female and male mice. Gating was performed as outlined in Fig. S2A. Single cell preparations of lungs were stained for granulocytes, characterized by surface expression of LyG6^+^, F4/80^-^, Ly6C^+^, CD11c^-^ ((A), (B)), monocytes/macrophages (MFs) distinguished by Ly6G^+^, F4/80^+^, Ly6C^-^, CD11c^+^ ((C), (D)), and dendritic cells (DCs) showing Ly6G^-^, F4/80^+^, Ly6C^+^ CD11c^-^ ((E), (F)). Total CD45^+^ cells were defined as 100% for granulocytes ((B), (D), (F)) and the total percentage of MFs and DCs was set as 100% to calculate their ratio ((A), (C), (E)). Data are shown as whisker box plots providing medians, IQR, Q1, Q3, minimum, and maximum data values. When samples were normalized ((A), (C), (E)), sham is set as 1. Statistical inference was performed using linear mixed-effects models. Results of the omnibus tests and corresponding pairwise comparisons with BH-adjusted P values are provided in Supplementary Tables S5–S8. Light red, young female; blazing red, aged female; light blue, young male; royal blue, aged male mice; dotted green line (= sham) was set as 1. IT, intratracheal; LPS, lipopolysaccharide.

**Fig. S11 | Flow cytometric profiling of the myeloid immune cell subtypes in the spleen of young and aged sham mice of both sexes.** Flow cytometric analyses of myeloid immune cells derived from the spleen of sham and LPS-treated young (8-12 weeks) and aged (15 -18 months) female and male mice. Gating was performed as outlined in Fig. S2A. Single cell preparations of spleens were stained for granulocytes, characterized by surface expression of LyG6^+^, F4/80^-^, Ly6C^+^, CD11c^-^ ((A), (B)), monocytes/macrophages (MFs) distinguished by Ly6G^+^, F4/80^+^, Ly6C^-^, CD11c^+^ ((C), (D)), and dendritic cells (DCs) showing Ly6G^-^, F4/80^+^, Ly6C^+^ CD11c^-^ ((E), (F)). Total CD45^+^ cells were defined as 100% for granulocytes (B), and the total percentage of MFs and DCs was set as 100% to calculate their ratio ((D), (F)). Data are shown as whisker box plots providing medians, IQR, Q1, Q3, minimum, and maximum data values. When samples were normalized ((A), (C), (E)), sham is set as 1. Statistical inference was performed using linear mixed-effects models. Results of the omnibus tests and corresponding pairwise comparisons with BH-adjusted P values are provided in Supplementary Tables S5–S8. Light red, young female; blazing red, aged female; light blue, young male; royal blue, aged male mice; dotted green line (= sham) was set as 1. IT, intratracheal; LPS, lipopolysaccharide.

**Fig. S12 | Flow cytometric profiling of the myeloid immune cell subtypes in the mediastinal lymph node of young and aged sham mice of both sexes.** Flow cytometric analyses of myeloid immune cells derived from the lymph node of sham and LPS-treated young (8-12 weeks) and aged (15 -18 months) female and male mice. Gating was performed as outlined in Fig. S2A. Single cell preparations of mediastinal lymph nodes were stained for granulocytes, characterized by surface expression of LyG6^+^, F4/80^-^, Ly6C^+^, CD11c^-^ ((A), (B)), monocytes/macrophages (MFs) distinguished by Ly6G^+^, F4/80^+^, Ly6C^-^, CD11c^+^ ((C), (D)), and dendritic cells (DCs) showing Ly6G^-^, F4/80^+^, Ly6C^+^ CD11c^-^ ((E), (F)). Total CD45^+^ cells were defined as 100% for granulocytes (B), and the total percentage of MFs and DCs was set as 100% to calculate their ratio ((D), (F)). Data are shown as whisker box plots providing medians, IQR, Q1, Q3, minimum, and maximum data values. When samples were normalized ((A), (C), (E)), sham was set as 1. Statistical inference was performed using linear mixed-effects models. Results of the omnibus tests and corresponding pairwise comparisons with BH-adjusted P values are provided in Supplementary Tables S5–S8. Light red, young female; blazing red, aged female; light blue, young male; royal blue, aged male mice; dotted green line (= sham) was set as 1. IT, intratracheal; LPS, lipopolysaccharide.

**Fig**. **S13 | Flow cytometric profiling of the lymphoid immune cell subpopulations in the blood of young and aged mice of both sexes.** Flow cytometric analyses of lymphoid immune cells derived from the blood of sham and LPS-treated young (8-12 weeks) and aged (12-15 months) female and male mice. Gating was performed as outlined in Fig. S2B. Single cell preparations of blood were stained for NK cells (NK1.1), characterized by surface expression of NK1.1 ((A), (B)), B cells distinguished by CD19^+^, NK1.1^-^ 1 ((C), (D)), and T cells showing CD3ε^+^, CD19^-^, NK1.1^-^ ((E), (F)). T helper (T_h_) cells were defined expressing CD4^+^CD3ε^+^CD8^-^CD19^-^NK1.1^-^ ((G), (H)) and cytotoxic T cells by surface staining of CD8^+^CD3ε^+^CD4^-^CD19^-^NK1.1^-^ ((I), (J)). Total CD45^+^ cells were defined as 100% for NK cells (B), B cells (D) and T cells (F). CD4^+^ and CD8^+^ cells were determined considering all CD3^+^ T cells set as 100% ((H), (J)). Data are shown as whisker box plots providing medians, IQR, Q1, Q3, minimum, and maximum data values. When samples were normalized ((A), (C), (E), (G), (I)), sham was set as 1. Statistical inference was performed using linear mixed-effects models. Results of the omnibus tests and corresponding pairwise comparisons with BH-adjusted P values are provided in Supplementary Tables S5–S8. Light red, young female; blazing red, aged female; light blue, young male; royal blue, aged male mice; dotted green line (= sham) was set as 1. IT, intratracheal; LPS, lipopolysaccharide.

**Fig. S14 | Flow cytometric profiling of the lymphoid immune cell subpopulations in the spleen of young and aged mice of both sexes following IT LPS treatment.** Flow cytometric analyses of lymphoid immune cells derived from the spleen of sham and LPS-stimulated young (8-12 weeks) and aged (15-18 months) female as well as male mice. Gating was performed as outlined in Fig. S2B. Single cell preparations of spleen were stained for NK cells (NK1.1), characterized by surface expression of NK1.1 ((A), (B)), B cells distinguished by CD19^+^, NK1.1^-^ ((C), (D)), and T cells showing CD3ε^+^, CD19^-^, NK1.1^-^ (€, (F)). T helper (T_h_) cells were defined expressing CD4^+^CD3ε^+^CD8^-^CD19^-^NK1.1^-^ ((G), (H)) and cytotoxic T cells by surface staining of CD8^+^CD3ε^+^CD4^-^CD19^-^NK1.1^-^ ((I), (J)). Total CD45^+^ cells were defined as 100% for NK cells (B), B cells (D) and T cells (F). CD4^+^ and CD8^+^ cells were determined considering all CD3^+^ T cells set as 100% ((H), (J)). Data are shown as whisker box plots providing medians, IQR, Q1, Q3, minimum, and maximum data values. When samples were normalized ((A), (C), (E), (G), (I)), sham was set as 1. Statistical inference was performed using linear mixed-effects models. Results of the omnibus tests and corresponding pairwise comparisons with BH-adjusted P values are provided in Supplementary Tables S5–S8. Light red, young female; blazing red, aged female; light blue, young male; royal blue, aged male mice; dotted green line (= sham) is set as 1. IT, intratracheal; LPS, lipopolysaccharide.

**Fig. S15 | Flow cytometric profiling of the lymphoid immune cell subpopulations in the mediastinal lymph node of young and aged mice of both sexes following IT LPS treatment.** Flow cytometric analyses of lymphoid immune cells derived from the lymph node of sham and LPS-stimulated young (8-12 weeks) and aged (15-18 months) female as well as male mice. Gating was performed as outlined in Fig. S2B. Single-cell preparations of the mediastinal lymph node were stained for NK cells (NK1.1), characterized by surface expression of NK1.1 ((A), (B)), B cells distinguished by CD19^+^, NK1.1^-^ ((C), (D)), and T cells showing CD3ε^+^, CD19^-^, NK1.1^-^ ((E), (F)). T helper (T_h_) cells were defined expressing CD4^+^CD3ε^+^CD8^-^CD19^-^NK1.1^-^ ((G), (H)) and cytotoxic T cells by surface staining of CD8^+^CD3ε^+^CD4^-^CD19^-^NK1.1^-^ ((I), (J)). Total CD45^+^ cells were defined as 100% for NK cells (B), B cells (D) and T cells (F). CD4^+^ and CD8^+^ cells were determined considering all CD3^+^ T cells set as 100% ((H), (J)). Data are shown as whisker box plots providing medians, IQR, Q1, Q3, minimum, and maximum data values. When samples were normalized ((A), (C), (E), (G), (I)), sham was set as 1. Statistical inference was performed using linear mixed-effects models. Results of the omnibus tests and corresponding pairwise comparisons with BH-adjusted P values are provided in Supplementary Tables S5–S8. Light red, young female; blazing red, aged female; light blue, young male; royal blue, aged male mice; dotted green line (= sham) was set as 1. IT, intratracheal; LPS, lipopolysaccharide.

**Fig. S16 | Flow cytometric determination of FoxP3 cells in blood, spleen, and mediastinal lymph node of young and aged sham and LPS mice of both sexes.** Flow cytometric analyses of regulatory T cells (T_reg_) derived from blood, spleen, and mediastinal lymph node of sham and LPS stimulated young (8-12 weeks) and aged (15-18 months) female as well as male mice. Gating was performed as outlined in Fig. S2C. Single-cell preparations of blood ((A), (B)), spleens ((C), (D)), and lymph nodes ((E), (F)) were stained for CD3ε, CD4, and FoxP3 to identify T_regs_ (CD3ε^+^CD4^+^FoxP3^+)^. Total CD4^+^ cells were defined as 100% ((B), (D), (F)). FoxP3^+^ T cells were calculated accordingly. Data are shown as whisker box plots providing medians, IQR, Q1, Q3, minimum, and maximum data values. When samples were normalized ((A), (C), (E)), sham was set as 1. Statistical inference was performed using linear mixed-effects models. Results of the omnibus tests and corresponding pairwise comparisons with BH-adjusted P values are provided in Supplementary Tables S5–S8. Light red, young female; blazing red, aged female; light blue, young male; royal blue, aged male mice; dotted green line (= sham) was set as 1. IT, intratracheal; LPS, lipopolysaccharide.

**Fig. S17 | Principal component analysis (PCA) of pulmonary data derived from a preclinical murine ALI model.** PCA analyses of z-scores of lung (A) immune cell subpopulations, (B) mRNA expression, and (C) protein expression derived from female young (8-12 weeks) sham (light red triangle) and LPS (light red filled circle) as well as aged (12-15 months) sham (blazing red triangle) and LPS (blazing red filled circle) mice. Also, results from male young (8-12 weeks) sham (light blue triangle) and LPS (light blue filled circle) as well as aged (12-15 months) sham (royal blue triangle) and LPS (royal blue filled circle) mice were included.

Fig. S18

(A)	(B)

(C)

(D)

Bioplot – cell subtypes

(E)

Bioplot – genes

(F)

Bioplot – proteins

**Fig. S18 | Validation of PCA analysis.** For each data set ((A) cells, (B) genes, (C) proteins), the variance explained by each PC was calculated and the cumulative variance of the eight PCs was evaluated. Scree plots and the most relevant 5 or 10 factors, respectively, for PC1 and PC2 were provided. PCA results were visualized using Biplots (upper panels of (D) cells, (E) genes, (F) proteins) showing the top 5 contributing features of PC1. Group-specific differences were visualized using gradient-coloured scatter plots (middle panels of (D) cells, (E) genes, (F) proteins) and heat maps of z-scores (lower panels of (D) cells, (E) genes, (F) proteins).
